# Tailored Extracellular Vesicles: Novel Tool for Tissue Regeneration

**DOI:** 10.1155/2022/7695078

**Published:** 2022-07-29

**Authors:** Linli Li, Peipei Wu, Hui Qian, Wenrong Xu, Hui Shi, Jiajia Jiang

**Affiliations:** ^1^Aoyang Institute of Cancer, Affiliated Aoyang Hospital of Jiangsu University, 279 Jingang Road, Suzhou, 215600 Jiangsu, China; ^2^Zhenjiang Key Laboratory of High Technology Research on EVs Foundation and Transformation Application, School of Medicine, Jiangsu University, 301 Xuefu Road, Zhenjiang, Jiangsu, China

## Abstract

Extracellular vesicles (EVs) play an essential part in multiple pathophysiological processes including tissue injury and regeneration because of their inherent characteristics of small size, low immunogenicity and toxicity, and capability of carrying a variety of bioactive molecules and mediating intercellular communication. Nevertheless, accumulating studies have shown that the application of EVs faces many challenges such as insufficient therapeutic efficacy, a lack of targeting capability, low yield, and rapid clearance from the body. It is known that EVs can be engineered, modified, and designed to encapsulate therapeutic cargos like proteins, peptides, nucleic acids, and drugs to improve their therapeutic efficacy. Targeted peptides, antibodies, aptamers, magnetic nanoparticles, and proteins are introduced to modify various cell-derived EVs for increasing targeting ability. In addition, extracellular vesicle mimetics (EMs) and self-assembly EV-mimicking nanocomplex are applied to improve production and simplify EV purification process. The combination of EVs with biomaterials like hydrogel, and scaffolds dressing endows EVs with long-term therapeutic efficacy and synergistically enhanced regenerative outcome. Thus, we will summarize recent developments of EV modification strategies for more extraordinary regenerative effect in various tissue injury repair. Subsequently, opportunities and challenges of promoting the clinical application of engineered EVs will be discussed.

## 1. Introduction

Extracellular vesicles (EVs) are nanosized biogenic particles which can be isolated from multiple types of cells, tissues, and body fluids. They contain multiple bioactive molecules such as nucleic acids, proteins, lipids, and metabolites and can mediate intercellular communication in a short-and long-distance way. In addition, their characteristics including nanosize, low immunogenicity and toxicity, and easy crossing of physiological barrier are of great interest [1–3]. EVs derived from various cells play a vital role in tissue regeneration because of biologically active molecules inherited from parent cells and their inherent characteristics. For instance, accumulating studies have demonstrated that EVs derived from mesenchymal stem cells (MSCs) participate in accelerating wound healing, promoting cartilage tissue and bone tissue and neuronal regeneration, attenuating liver and renal damage, and strengthening myocardial regeneration [4–9], the mechanisms of which mainly include antiapoptosis, the promotion of cell proliferation and differentiation and migration, angiogenesis, and immune regulation.

However, the limited therapeutic efficacy, poor targeting ability, low yield for production of naive EVs, and rapid clearance from the body partly restrict EV clinical application. Growing evidences have suggested that EVs can be modified to overcome the obstacles mentioned above, and the modified strategies predominantly involve cargo loading, targeting modification, the use of EMs which are synthetic extracellular vesicle-mimetic nanovesicles, self-assembly EV like nanocomplex, and combined application of EVs with biomaterials. The direct injection of therapeutic agents like nucleic acids, peptides, proteins, and drugs remains a challenge because these therapeutic agents may have drawbacks including short-life, rapidly being cleared from the body, complications to nontreatment tissue, and inefficient ability to cross physiological barriers like blood-brain barrier (BBB). Increasing studies have demonstrated that encapsulating therapeutic agents into EVs through indirectly incorporating cargos into donor cells or directly packaging cargos into EVs is a feasible strategy to strengthen therapeutic outcome owing to extraordinary inherent characteristics of EVs [10, 11]. And currently, the clinical assessments of EVs secreted from MSCs are undergoing for nanosized delivery in the field of regenerative medicine [12]. Besides, the biodistribution of EVs also affect their repairing effects. While EVs possess an inherent targeting ability compared with other synthetic cargos carrier like liposomes, the targeting ability of EVs are associated with the sources, the membrane components, administration mode of EVs, and pathophysiological condition of host [13]. Increasing studies have demonstrated that better therapeutic effects can be achieved through presenting surface ligands on EVs to enhance tissue and organ targeting ability. The ligands may include targeting peptides, antibodies, several proteins, and aptamers. Furthermore, EVs encapsulating magnetic nanoparticles like Fe_3_O_4_ and superparamagnetic iron oxide nanoparticles (SPION) and macrophage cell membrane-fused EMs nanovesicles (MF-NVs) are also used to enhance EV targeting ability. As for the production of EVs, Shao et al. have shown that only 1-4 *μ*g of EV proteins are produced from 1 × 10^6^ MSC cells per day, indicating that the EVs secreted from MSC cells are limited [14]. How to enhance EV yield is necessary for future application in clinic. Some strategies like pH variations or low-oxygen conditions and chemical stimuli are employed to improve EV yield. However, the effect in a long-term brought by these approaches on the physiological properties of EVs need to be further determined [12]. EMs and self-assemble EV like nanocomplex are a feasible method to overcome the difficulties in poor yield of EVs. The retention time of EVs in the body is another consideration for better regenerative outcome. In recent years, EV-based tissue engineering has attracted increasing attention in regeneration medicine because the combination of EVs and biomaterials including hydrogel, scaffolds, and dressings are capable of achieving sustained delivery of EVs, recruit endogenous cells to proliferate, provide space for cell growth, and then result in synergistically enhanced tissue repair effects.

**Figure 1:**
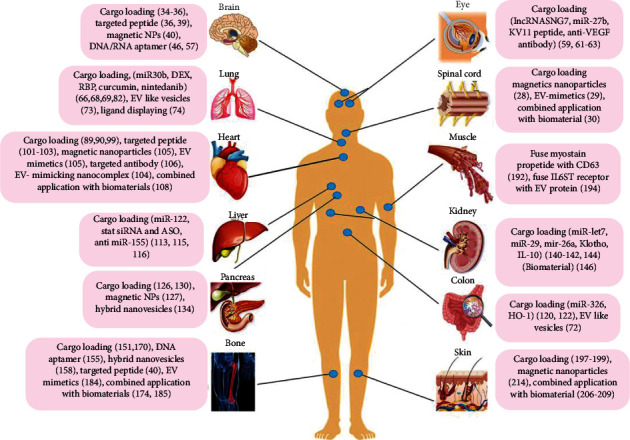
Engineered EV enhances major tissue and organ repairs. The modification approaches of EVs in the therapy of tissue and organ injury mainly involve four aspects: cargo loading, targeting modification, EV mimetics and EV-mimicking nanocomplex, and the combined application of EV with biomaterials.

In this review, we highlight the recent advanced strategies of engineering EVs (loading therapeutic molecules and drugs into EVs, strengthening EV targeting ability, designing EV mimetics and EV-mimicking nanocomplex, combining EVs with biomaterials) in various tissue (neural tissue, eye, lung, heart, liver, intestine, pancreas islet, renal, bone, muscle, and skin) (Figure 1). In addition, we provide a perspective on the prospects of modified EVs. We look forward to helping better understand key challenges and opportunities in the application of modified EVs in regeneration medicine.

## 2. Extracellular Vesicles

Extracellular vesicles are endosome-derived vesicles which can be released by nearly all cells [15, 16]. According to their size, biogenesis, and contents, EVs are mainly classified into three subtypes: exosomes, microvesicles (MVs), and apoptotic bodies. Exosomes are ranged from 30 to 180 nm in size, and the components of exosomes mainly include structural proteins (such as HSP90, Alix, TSG101, CD9, CD63, TSPAN29, and flotillin), specific proteins (such as MCH-I, MCH-II, CD80, CD86, FasL, and TGF-*β*), microRNAs, mRNA, and other noncoding RNA [17, 18]. In brief, the exosome biogenesis involves multiple processes in which plasma membrane is internalized to form early endosome followed by transition to late endosomes which is also called multivesicular bodies (MVBs) and intraluminal vesicle (ILVs) formation within the endosome. Finally, MVBs fuse with plasma membrane and exosomes are released from the ILVs.

MVs (50 to 1000 nm) are formed by the budding of plasma membrane, and MV markers are characterized by integrins, selectins, and CD40 ligand, and their components include mRNA, miRNA, other noncoding RNAs, and cytoplasmic and membrane proteins [15, 19]. Apoptotic bodies (500 to 2000 nm) contain part of both nuclear and fragments and cell organelles released by cells under the process of programmed death [20].

## 3. Neurological Disorders

### 3.1. Spinal Cord Injury

Spinal cord injury (SCI), as the second major contributor of paralysis, leads to temporary or permanent loss of sensory and motor functions and results in massive cell death, inflammation, vascular injury, severe oxidative stress, and glial scar formation at the lesion area [21, 22]. EVs derived from MSCs, M2 macrophage, neural stem cells, and neurons have been reported to have potential to treat SCI via inhibiting neuronal apoptosis, degeneration, inflammatory response, and glial scar formation and promoting axonal regeneration and angiogenesis. Furthermore, modified EVs similarly are employed in the therapy of SCI. miRNAs such as miR-124-3p [23], miR-26a [24], lncRNAs like lncGm37494 [25], and plasmid cDNA such as GIT1 [26] and sonic hedgehog [27] are transfected to donor cells, and then modified EVs are isolated to treat SCI (Table 1). As a result, modified EVs loaded with therapeutic agents greatly enhanced SCI recovery compared with the control group. Although the method of transfecting donor cells to load cargos is simple and widely used, it may affect other molecules in donor cells, and the loading efficiency is limited. Besides gene agents, EVs can also be used as a drug delivery tool to overcome the shortcomings of SCI therapy-related drugs such as short half-life, inefficient capacity to cross BBB, and easy clearance. Berberine-loaded M2 macrophage-derived EVs can efficiently cross BBB to target the injured spinal cord due to macrophage-derived EVs with inherent ability to target inflammation, and subsequently, this engineered EVs showed a decent anti-inflammatory and antiapoptotic effect [10]. Moreover, Kim et al. constructed iron oxide nanoparticle- (IONP-) encapsulated EM nanovesicles (NV-IONP) from IONP-treated human MSCs (hMSCs). IONP with magnetic guidance not only endowed NVs with targeting ability but also increase the contents of therapeutic growth factors in NV [28]. Similarly, taking the advantages of EMs and macrophage membranes, macrophage membrane-fused EM nanovesicles (MF-NVs) were generated to improve targeting ability and therapeutic efficiency [29]. Apart from systematic administration, topical transplantation of MSC-derived EVs which were immobilized in a peptide-modified adhesive hydrogel is also employed to comprehensively mitigate SCI microenvironment [30]. Furthermore, multifunctional scaffold capable of retaining paclitaxel delivered EVs within scaffold is designed, and this scaffold has capability in recruiting endogenous neural stem cells to the injured site, enhancing neural regeneration and decreasing scar deposition [21].

### 3.2. Stroke

**Figure 2:**
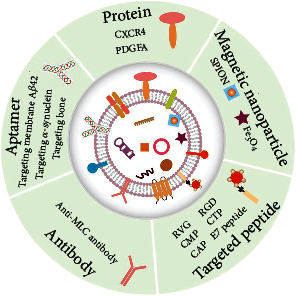
The strategies of engineering EVs to increase their targeting ability. The targeting modification mainly involves the utilization of targeted peptide (RVG, RGD, CMP, CTP, CAP, and E7 peptide), magnetic nanoparticles (SPION, Fe_3_O_4)_, protein (CXCR4, PDGFA), aptamer (Targeting membrane A*β*42, *α*-synuclein and bone), and antibody (anti-MLC).

Stroke is one of the leading causes of death and disability worldwide [31]. Currently, there is no drugs available for the cure of stroke. Mounting evidences have demonstrated that EVs from MSCs remote ischemic preconditioning cells, endothelial progenitor cells, M2 microglia, and astrocytes are involved in the treatment of stroke mainly through enhancing angiogenesis and neurogenesis. Furthermore, modified EVs from cells transfected therapeutic miRNAs such as miR-126 [32], miR-223-3p [33], miR-17-92 [34], circular RNAs like circSHOC2 [35], NGF mRNA [36], and plasmid cDNA such as CCR2 [37] and CXCR4 [38] have showed enhanced therapeutic effects in contrast with natural EVs. Although loading bioactive molecules into EVs did improve therapy efficacy, increasing targeting ability of EVs to the injured brain is another consideration. Recently, c(RGDyK) peptide (Figure 2) is conjugated on the surface of EVs *via* bio-orthogonal copper-free click chemistry method, and then cholesterol modified miR-210 is directly delivered into EVs (RGD-EV-miR-210) *via* incubation. As a result, RGD-EV-miR-210 is demonstrated to be promising NVs which are capable of targeting to the injured brain and promoting microvascular formation, eventually alleviating ischemia brain symptoms. Meanwhile, the method of directly loading cargos *via* incubation with cholesterol-miR-210 seems to be better in keeping the integrity of EVs compared with several approaches like electroporation and sonication [39]. Furthermore, the click chemistry method is quicker and simpler in contrast with fusing targeted peptide with EV membrane protein [2]. Similarly, Yang et al. generated NGF-EV-RV by cotransfecting RVG-LAMP2B and pCI-neo-NGF plasmids into donor cells, and NGF-EV-RV not only showed efficient targeting ability to injured region but also decreased inflammation, promoted cell survival, and increased the population of doublecortin-positive cells [36]. Apart from targeting peptides, magnetic NVs (MNVs) are also fabricated *via* incorporating IONPs into MSC, and then MNVs are made by means of extrusion. IONP enables MNVs to possess excellent targeting ability, upregulate the expression of grow factors, significantly decrease infarction volume, and improve motor function of the brain [40]. Edaravone (Edv) is a type of clinical drugs primarily used for the treatment of brain infarction. It is reported that macrophage-derived EVs encapsulating Edv are capable of releasing drugs at ischemic region, thereby improving bioavailability and neuroprotective effect of Edv and decreasing ischemic cerebral infarction [11]. In addition, Tian et al. constructed a simple, quick, and efficient drug delivery system *via* conjugating c(RGDyK) peptide to the surface of EV (cRGD-EV), and curcumin was loaded onto the cRGD-EV (cRGD-EV-cur). cRGD-EV-cur significantly inhibited the inflammatory response and cellular apoptosis in the injury region [41]. In clinic, numerous drugs are being “trialed” to treat stroke, loading these drugs to EVs with targeting ability to injury brain hold great potential in augmenting drug therapeutic effect.

### 3.3. Parkinson's Disease

Alpha-synuclein (*α*-syn) aggregates play a key role in the pathogenesis of Parkinson's disease (PD) which is the second most prevalent neurodegenerative disorder worldwide [42, 43]. Thus, decreasing *α*-syn expression is an essential target for the treatment of PD. In recent years, the EV-based delivery system of gene to downregulate the *α*-syn expression or reduce the *α*-syn pathological aggregates has emerged as a crucial tool for the therapy of PD. Specifically, shRNA minicircles (shRNA-MCs), a type of double-stranded DNA vectors which are smaller than plasmid and have longer term gene silencing function compared with siRNA, were loaded into RVG modified EVs by electroporation. This engineered strategy achieved specific delivery of shRNA-MCs into the brain and decreased *α*-syn aggregation [43]. Notably, it is necessary to evaluate the EV integrity and the change of shRNA-MCs when it comes to the method of electroporation. In recent years, there emerges a novel agent, aptamer, which is also called “chemical antibody” with targeted therapy and diagnostic ability. More importantly, aptamer with high specificity and affinity are low toxic, low immunogenic, smaller, and more extraordinary in tissue penetration in contrast with antibody [44, 45]. DNA aptamers that specifically recognize *α*-syn and retard preformed fibril recruiting endogenous a-syn into pathologic aggregates were packaged into EVs which were modified with the neuron-specific RVG peptide as well, and then the aptamer-loaded RVG-EVs greatly decreased pathological *α*-syn aggregates and improved motor dysfunction [46]. Aptamer-loaded RVG-EVs maybe a hopeful candidate for PD treatment. Apart from the application of shRNA-MCs and aptamer, bone marrow MSC- (BMSCs-) derived EV-mediated delivery of antisense oligonucleotides (ASO) that selectively ameliorate the *α*-syn expression also provide an alternative for the treatment of PD [47]. Moreover, catalase-loaded EVs secreted by monocytes macrophages and dopamine-loaded blood derived EVs have neuroprotective effects on PD [48, 49]. Based on gene therapy, Liu et al. recently constructed a multifunctional core-shell delivery platform named “nanoscavenger” to achieve synergetic therapeutic effect of gene and chemical drugs, which is composed of immature dendritic cell- (imDC-) derived EVs modified with RVG peptide, curcumin, phenylboronic acid-poly (2-(dimethylamino) ethyl acrylate) nanoparticle, and small interfering RNA targeting SNCA (REV-C/ANP/S). Among these components, core part consists of C/ANP/S which loaded simultaneously siRNA targeting SNCA (siSNCA) with the capability in attenuating the *α*-syn protein expression and curcumin with the ability in reducing the existing *α*-syn aggregates. RVG modified imDC-EVs served as the shell which not only have functions in crossing BBB, targeting lesion area, increasing the drugs bioavailability, and decreasing systematic toxicity but also exhibit ability in clearing immune activation because of the existence of imDC-EVs [50]. This platform not only successfully delivered gene-chem cargos to lesion region in a collaborative and targeted way but also increased loading efficiency compared with direct packaging of cargos into EVs.

### 3.4. Alzheimer's Disease

Alzheimer's disease (AD) is the most common type of dementia, and growing evidences suggest that AD is associated with deposition of the amyloid-beta protein (A*β*) and neuronal fiber tangles [51]. Modified EVs mainly involve gene and drug loading and targeting peptide modification in the treatment of AD. Specifically, EVs packaging miR-29 secreted from cells transfected with miR-29 have enhanced therapeutic effects on spatial learning and memory in animals [52]. Natural EVs passively target and accumulate in some specific organs such as the liver and spleen depending on its inherited property, therefore decreasing its targeting efficiency in other organs and weakening drug therapeutic efficacy in disease treatment, especially in central nervous disease therapy. And a variety of therapeutic EVs were modified by specific recognizable ligands, aptamer, etc. to achieve drug targeted delivery. In recent years, plasma-derived EVs were found to possess the unique properties including the innate ability of crossing the BBB and immunologic inertia; more importantly, plasma-derived EVs not only improve the bioavailability of drugs but also can achieve drug brain targeted delivery across BBB because some peptides inherited by plasma-derived EVs can specifically bind to receptors in the brain, thereby increasing the accumulation of EVs in the brain [53–55]. Therefore, considering that quercetin (Que) is promising in strengthening cognitive ability and the capability of plasma derived EVs in achieving drug brain targeted delivery and improving drug bioavailability, Que is loaded into plasma EVs with inherent brain targeting capability (EV-Que), and then Que endowed EV-Que with neuroprotective effects *via* inhibiting CDK5-mediated Tau phosphorylation [55]. EVs derived from curcumin primed cells are also reported to be capable of reliving the symptoms of AD [51]. In light of the capability of MSC-derived EVs to treat AD, Cui et al. proposed to use RVG peptide to modify EVs to realize targeting to cortex and hippocampus region and then significantly improved learning and memory capabilities in mice [56]. Moreover, for alleviating symptoms of AD, RNA aptamers that selectively bind membrane A*β*42 were also packaged into EVs to reduce reactive oxygen species production [57].

## 4. Eye Diseases

In proliferative diabetic retinopathy (PDR), endothelial-to-mesenchymal transition (EndoMT) can lead to the occurrence of pathological fibrosis [58]. It has been demonstrated that EndoMT in DR can be suppressed by EVs collected from MSC overexpressing lncRNA SNHG7, thereby delaying DR progression [59]. Moreover, miR-486-3p modified EVs secreted from BMSCs also enhanced the treatment efficacy of DR through inhibiting cell oxidative stress, inflammation, and apoptosis and promoting proliferation *via* TLR4/NF-*κ*B axis [60]. Subretinal fibrosis, the end stage of neovascular age-related degeneration which can lead to severe and irreversible vision loss and subretinal fibrosis, is associated with epithelial-mesenchymal transition (EMT). Recently, Li et al. found that human umbilical cord MSC- (HucMSCs-) derived EVs encapsulating miR-27b can efficiently suppress EMT *via* miR-27b/ HOXC6 axis [61]. Suppressing abnormal blood vessel growth is an important aspect for the treatment of some ocular diseases such as retinopathy of prematurity (ROP) and PDR. KV11, an 11-amino acid peptide, shows an anti-angiogenesis effect in vitro and in several animal models. Meanwhile, considering that EVs may preferentially home to the cells from which they are secreted, KV11 was linked to epithelial cell- (EC-) derived EVs *via* an anchoring peptide, CP05 which can specifically bound to EV membrane protein CD63 (Table 2). KV11-EVs showed more excellent therapeutic outcome. Specifically, KV11-EVs were more efficiently delivered to the blood vessels of the mouse retina, more effective in inhibiting neovascularization and vessel leakage, and showed stronger effect on inhibiting VEGF-downstream signaling than KV11 alone. And the therapeutic effect of KV11-EVs was comparable to the intravitreal injection of VEGF-trap. More importantly, the administration mode of retroorbital injection in this system is safer and less invasive compared with intravitreal injection with single KV11 [62]. KV11-EVs may also be used for the therapy of other diseases associated with pathological angiogenesis, and it would be possible to add several other antiangiogenesis peptides to modify EVs. Taking advantage of anti-inflammation property of EVs derived from regulatory T cells (Treg-EVs) and the ability of VEGF antibody in suppressing VEGF activity, Tian et al. engineered Treg-EVs *via* conjugating VEGF antibodies to the surface of Treg-EVs to strengthen the repair outcome in choroidal neovascularization (CNV)[63]. As mentioned above, aptamer has more advantages over antibody; thus, we believe that it would be interesting to replace antibody with aptamer notwithstanding the synthesis of aptamer is challenging.

## 5. Lung-Related Disease

### 5.1. Acute Lung Injury

Recently, it is reported that EVs from clinical-grade allogenic adipose-derived MSCs (ADSCs) are efficient in the therapy of acute respiratory distress syndrome and healthy volunteers who received the inhalation of EVs have no serious side effects [64]. It demonstrated that EVs may serve as a promising candidate for the therapy of lung-related diseases in clinic. Acute lung injury (ALI) represents a clinical disorder that may associate with inflammation response, oxidative stress, and fibrosis progression [65]. For more pronounced therapeutic effect, EVs were engineered by overexpressing MSCs with miR-30b-3p to relive inflammation more effectively in ALI mice [66]. Dexamethasone (DEX) has been employed to decrease inflammation in clinic; however, the side effects brought by DEX cannot be ignored. Platelet-derived extracellular vesicles (PEVs) with similar surface glycoproteins and transmembrane proteins of platelets include CD40L, glycoproteins Iba, aIIb, and VI, and P-selectin, which endows PEVs with the ability to recognize and targeted to the inflamed tissue [67, 68]. Therefore, DEX was loaded into PEVs (DEX–PEVs) via incubation to achieve targeted DEX delivery, and this strategy demonstrated a better therapeutic outcome to reduce hyperinflammation in the affected lungs and recover blood oxygen saturation. Moreover, the same dose DEX–PEV treatment in pneumonia mice significantly reduced side effects including abnormal behavioral symptoms of anxiety and irritability in contrast with single use of DEX [68] (Table 3). In addition to using the property of natural EVs to achieve targeted delivery of therapeutic agents to injured lung, RAGE-binding peptide (RBP) with lung-targeting ability and anti-inflammation effect is displayed on the surface of EVs *via* fusing with LAMP2B. And then curcumin with hydrophobic peculiarity and ability in decreasing reactive oxygen species is loaded into EVs as well. EVs simultaneously carrying RBP and curcumin succeed in significantly reliving ALI symptom [69].

### 5.2. Coronavirus Disease 2019

Coronavirus disease 2019 (COVID-19) patient in serious conditions is usually caused by SARS-CoV-2-mediated severe cytokine storm, which contributes to a tissue damage including apoptosis and necrosis and damage to alveolar epithelial cells and vascular ECs, and sustained lung invasion by continuous infiltration of immune cells [70]. Multiple clinical trials are evaluating the efficacy of and MSC-derived EVs in the remission of COVID-19 in critically ill patients. Furthermore, Jamalkhah et al. present an unprecedented strategy of modifying MSC-EVs with interfering RNAs which would hinder viral propagation, inflammation induction, and immune escape in already-infected cells and obstruct the viral particles' entrance to the uninfected cells and lung tissue to further enhance the antiviral immune [71]. Natural EV-like ginger-derived lipid vehicles (GDLVs) that is isolated from edible ginger plant with low toxicity and can be produced in large scale are nanoparticles similar to EVs containing proteins, lipids, and RNAs, in particular, small-sized RNA [72, 73]. MiRNA aly-miR396a-5p packed into GDLVs by incubation is delivered to the lung and effectively inhibits lung inflammation. Moreover, GDLVs encapsulating aly-miR396a-5p and rlcv-miRrL1-28-3p remarkably suppressed SARS-CoV-2-induced cytopathic effect by inhibiting the expression of Nsp12 and spike genes respectively [73]. In order to deliver potential antiviral agents into specific tissues, VSVG viral pseudotypin-based approach is constructed to load EV membranes with SARS-CoV-2 receptor-binding domain (RBD) of the viral spike protein which is the critical domain for SARS-CoV-2 attachment, fusion, and cellular entry. RBD modified EVs can accumulate in the lung because RBD can specifically recognize ACE2 receptor that is highly expressed at the surface of type 2 alveolar epithelial cells in the lung. Then, siRNA-GFP was packaged into EVs via electroporation, and the results showed that RBD successfully endowed EVs with cell targeting ability, and GFP signals significantly decreased in lung regions compared to the control group, implying that RBD-EVs with targeting capability may be a potential nanoparticle to deliver antiviral agents for treatment of SARS-CoV-2 for treatment of SARS-CoV-2 infection [74]. Besides, it is proposed that interrupting the interaction of ACE2 with viral spike protein and enrichment of ACE2 in EVs is promising in treating COVID-19. Considering that the EV-ACE2 is determined by protein palmitoylation which is essential for the membrane-targeting of ACE2 and their EV secretion, Xie et al. engineered EVs via fusing the S-palmitoylation-dependent plasma membrane (PM) targeting sequence with ACE2 (referred to as PM-ACE2-EVs), then the fusion of the PM targeting sequence increased the accumulation of ACE2 in EVs, and most of ACE2 are on the surface of PM-ACE2-EVs. The viral load of authentic SARS-CoV-2 was efficiently blocked by PM-ACE2-EVs, thus protecting host against SARS-CoV-2-induced lung inflammation [75].

### 5.3. Idiopathic Lung Fibrosis

There are few options for idiopathic lung fibrosis (IPF) treatment. Nowadays, only pirfenidone and nintedanib (NIN) are approved by FDA for the treatment for IPF, however, which are palliative and merely delay disease progression [76, 77]. It has been revealed that EVs from diverse cells have therapeutic potential to treat IPF disease [78–81]. In addition, EVs are further engineered for the therapy of IPF. Antifibrotic drug incapable of being successfully delivered to fibroblasts is a hurdle in the pulmonary fibrosis therapy. Recently, hybrid NVs composed of liposome loading with clodronate (CLD) and fibroblast-derived EVs are applied to deliver NIN. Among these components, liposome can increase NIN drug encapsulation efficiency. CLD and fibroblast-derived EVs enabled the EV biodistribution in the lung because the former reduces liver uptake of EVs *via* inducing apoptosis of macrophage at the liver, and thus hybrid NVs preferentially accumulate in the fibrotic lung; the latter shows efficient homing properties to their parent cells [82].

### 5.4. Allergic Asthma

Allergic asthma is an airway inflammatory disease characterized by bronchial hyperresponsiveness, mucosal edema, and airflow restriction [83]. It is reported that MSC-EVs can promote the immune-suppressive effect of Tregs by upregulating IL-10 and TGF-*β*1 from peripheral blood mononuclear cells of asthmatic patient [84]. In addition to the interaction of MSC-EVs with monocytes, the administration of MSC-EV by intranasal delivery expands lung IL-10-producing interstitial macrophages, thus contributing to protection against allergic asthma [85]. lncRNA Dnmt3aos plays a key role in M2 macrophages polarization which plays an important part in the occurrence of allergic asthma (AA). Based on this, a nanocomplex composed of smart silencer of Dnmt3aos (Dnmt3aos^smart silencer^) encapsulated polylactic-co-glycolic acid (PLGA) core and EV membrane of M2 macrophages shell was exploited. M2 macrophages EV membrane endowed nanocomplex with the ability to achieve targeted delivery of Dnmt3aos^smart silencer^ to injured lung tissue when PLGA improved the stability of nanocomplex thereby effectively delayed AA progression [86].

## 6. Cardiovascular-Related Diseases

### 6.1. Myocardial Infarction

Myocardial infarction (MI), namely, myocardial injury due to myocardial ischemia, is a leading cause of morbidity and mortality worldwide [87, 88]. EVs from MSCs, induced pluripotent stem cells (iPSCs), and immune cells like activated CD4^+^ T cells and DC, cardiac progenitor cells, and ECs have been demonstrated to play a pivotal role in cardioprotection after MI mainly *via* promoting cell proliferation and angiogenesis, ameliorating pyroptosis, and inhibiting inflammatory response and apoptosis. Furthermore, modified EV-mediated regeneration of MI mainly includes loading cargos into EVs by manipulation of parent cells, targeting peptide modification, EMs and EV-mimicking nanocomplex, and the combined use of EVs with biomaterials. For instance, therapeutic cargos like lncRNA KLF3-AS1 [89], miR-210 [90], miR-185 [91], miRNA-181a [92], Akt [93], SDF1 [94], TIMP2 [95], SIRT1 [96], MIF [97], CXCR4 [98], GATA-4 [99], and HIF-1*α* [100] are used to modify EVs and further enhanced therapeutic efficacy. In order to enhance cardiac-targeting of EVs, and several targeting peptides such as cardiomyocyte specific peptide (CMP), cardiac-targeting peptide (CTP), and ischemic myocardium-targeting peptide were fused with EV membrane protein LAMP2B (Table 4). The targeted peptide modified EVs exhibit increased cardiac retention and strengthened therapeutic efficacy in contrast with nontargeted modified EVs [101–103]. It is well known that low yields along with intricate purification processes of EVs are major hurdles for EVs clinical application. Yao et al. recently generated a type of MSC membrane-camouflaged EV-mimicking nanocomplex. In this nanocomplex, MSC membrane is coated on miR-21 loaded mesoporous silica nanoparticle (MSN) surface. This strategy fully took the advantages of MSC membrane which possesses intrinsic feature of protecting nanoparticles from immune clearance and possessing the capability in specific recognition to targeted cells. Meanwhile, MSN enabled high miRNA loading and effectively protects miR-21 from degradation [104]. Furthermore, apart from the treatment for nervous system-related diseases, IONP-MSC-derived NVs were also used in the therapy of MI [105]. Intriguingly, Liu et al. recently fabricated a “vesicle shuttle” which consists of a magnetic Fe_3_O_4_ core and a silica shell decorated with PEG corona which conjugated two types of antibodies (one bonded to EV surface membrane protein CD63, the other targeted to myosin-light-chain surface markers on injured cardiomyocytes). Thus, the magnetic-guided “vesicle shuttle” enabled efficient collection, targeted transport and release of EVs, and subsequently improved heart function after MI [106]. Various studies have demonstrated that EVs may be a promising therapeutic tool in MI, but it is reported that the retention of EVs is no more than 3 hours postmyocardial injection [107]. Recently, pluripotent stem cell-induced cardiomyocyte-derived EVs encapsulated hydrogel patch allowed for sustainable and slow release of packaged EVs in a rat model of acute MI and promoted infarcted heart recovery [108].

### 6.2. Atherosclerosis

Atherosclerosis is associated with EC dysfunction and injury which might be caused by inflammation and reactive oxygen species accumulation [109]. There are emerging studies suggesting that modified EVs also can be used in prevention or therapy of AS. MSC-derived miR-145-rich EVs inhibit HUVECs migration in vitro and reduced atherosclerotic plaque in vivo [110]. Furthermore, EVs from MSCs overexpressing miR-512-3p significantly inhibit ox-LDL-mediated EC damage by regulating the Keap1/Nrf2 signaling pathway [109] . Anti-inflammation is considered as a promising strategy for atherosclerosis treatment [111]. Wu et al. loaded an FDA approved hexyl 5-aminolevulinate hydrochloride (HAL) into M2 macrophage-derived EVs *via* electroporation. This kind of engineered EVs markedly strengthened the anti-inflammation effects and finally alleviated AS because M2 macrophage-derived EVs exhibit inherent excellent inflammation-tropism and anti-inflammation effects. More importantly, packaged HAL generates anti-inflammatory carbon monoxide and bilirubin, which further enhanced the anti-inflammation effects [111]. As mentioned above, EMs can be obtained to overcome the obstacles of low yields for EVs by techniques of extrusion. However, high loss caused by hanging on the filter membranes during extrusion remains a challenge that is needed to be optimized. In light of this, 5 freeze and thaw cycles (FT) of MSCs before serial extrusion have been proposed. FT/NVs were isolated using a tangential flow filtration (TFF) system in place of density gradient ultracentrifugation after serial extrusion. Because these processes were improved, FT/NVs with both high yield and high purity were obtained and FT/NVs successfully alleviated TNF-*α* induced inflammation as the same as NVs [112].

## 7. Digestive Diseases

### 7.1. Liver Injury

Currently, there is still no very effective antifibrosis therapy in clinical treatment. The cell-free treatment strategy represented by engineered EVs has brought new hope for the treatment of liver fibrosis. MiRNA (e.g., miR-122 and miR-181-5p) modified EVs by overexpressing ADSCs have a potential in treating liver fibrosis [113, 114]. STAT3 is closely associated with the pathogenesis of liver fibrosis. sIRNAs or ASO targeting STAT3 was packaged into clinical grade fibroblast-like MSC-derived EVs by electroporation significantly downregulated STAT3 levels as well as improved liver function in liver fibrosis mice [115]. Although EVs encapsulating these nucleotides did enhance the efficiency in prevention and treatment of liver fibrosis, the efficiency of strategies in loading cargos to EVs mentioned above is limited. Based on this, recent studies by Li et al. established a new engineering strategy for RNA cargos encapsulation by fusing CD9 with human antigen R (HuR) which is an RNA binding protein with capacity in interacting with AU rich elements (AREs) of RNAs. And fused CD9-HuR succeeded in enriching and packaging miR-155 inhibitor with AREs modification into EVs, which subsequently reduced miR-155 level in liver and significantly decreased CCL4 induced liver fibrosis. Moreover, CD9-HuR functioned EV can be utilized to load CRISPR/Cas9 which is difficult to be loaded into EVs by other approaches owing to its long length [116] (Table 5). Thus, CD9-HuR functioned EVs have great potential in RNA delivery of interest because of high loading efficiency. Knockdown of specific RNA is of great importance in the treatment of several diseases, to date, which is predominantly achieved by RNA interference (RNAi). Recently, EVs engineered with LAMP2B-HuR was developed to enrich specific RNA for lysosome degradation, which provide an alternative strategy for RNA degradation in cells particularly macrophage resistant to RNAi. That is to say, this system can be applied for macrophage-related disease therapy [117]. Here, the enhanced therapeutic efficacy has also been found from small molecule drug or cytokine preconditioning of MSC-derived EVs in acute liver injury models. Sun et al. found that melatonin preconditioned-ADSC-derived EVs exhibited better protection against hepatic ischemia-reperfusion injury [118]. IL-6-stimulated MSCs highly expressed miR-455-3p that can target PI3K signaling, which could attenuate macrophage infiltration and local liver damage and reduce the serum levels of inflammatory factors, thereby improving liver histology and systemic disorder [119].

### 7.2. Intestine Injury

Emerging studies suggest that gene or protein modified EVs played a vital role in promoting intestine injury repair. miR-326 directly packaged to MSC-EVs via Exo-Fect™ transfection reagent exhibits stronger therapeutic outcome on IBD compared with the control group [120]. Notably, the loading efficiency of commercial transfection reagents with potential toxicity is low in contrast with other methods like electroporation [121]. Oral administration of drugs gains most interest for IBD therapy. However, this administration mode remains challenging because drugs may be degraded in the set of gastrointestinal tracts, lack of targeting ability to injured intestine, and accompanied with several adverse effects. Recently, in addition to the treatment of COVID-19, GDLVs were also produced to encapsulate siRNA targeting CD98 which increased in intestine when IBD occurs. Orally administered GDLV packaging siRNA-CD98 can specifically target to colon tissues and decrease CD98 level. GDLVs hold great promise as drug carrier in IBD therapy because their production is more scalable and economical [72]. EVs derived from BMSCs transfected with heme oxygen-1 (HO-1) which then were transferred into intestinal epithelial cells resulting reduced cell apoptosis and inflammatory damage [122].

## 8. Diabetes and Its Related Complication

### 8.1. Diabetes

Diabetes mellitus (DM) is a metabolic disease, and type 1 and type 2 diabetes mellitus are common in clinic. The T1DM is mainly caused by the elimination of *β*-cells because of autoimmune destruction, while T2DM occurs because of decreased insulin sensitivity of peripheral tissues and a certain degree of pancreatic islet *β*-cells damage [123]. It has been demonstrated that MSC-EVs can delay T1DM progression *via* immune regulation, promotion of *β*-cell regeneration, and insulin secretion. Moreover, miR-26a in *β*-cells through circulating EVs reversed obesity-induced insulin resistance and hyperinsulinemia [124]. Meanwhile, there emerged several modified EVs in the treatment of diabetes. EVs transfected with an miR-133b mimic by the Exo-Fect [125] and M2 polarized bone marrow-derived macrophages derived EVs packaged with miR-690 [126] both enhanced glucose tolerance, insulin sensitivity, and thus provided a new insulin-sensitizing agent for the treatment of metabolic diseases (Table 6). In addition to EV-based gene therapy, BAY55-9837 peptide capable of inducing glucose-dependent insulin secretion however with shortcomings of short half-life, lack of targeting ability, and poor blood GLC response was loaded into EVs which were decorated with SPIONs, namely, BAY-EV-SPION. BAY-EV-SPION overcame shortcomings of single use of BAY55-9837 peptide, thereby significantly augmented insulin secretion [127].

### 8.2. Diabetic Nephropathy

Diabetic nephropathy (DN), one of diabetes complications, is considered as the most severe microvascular complication after DM [128]. Modified EVs in the therapy of DN mainly focused on isolating EVs from MSCs transfected with miRNAs. For instance, BMSC-EVs encapsulating miRNA-let-7a, ADSC-EVs mediated the delivery of miR-215-5p, human urine-derived MSC-EVs overexpressing miR-16-5p, and ADSC-EVs carrying miR-125a further protect against diabetic nephropathy by targeting USP22, ZEB2, VEGFA, and HDAC1 respectively [129–132].

### 8.3. Diabetic Peripheral Neuropathy

Engineered EVs were also employed in the field of diabetic peripheral neuropathy (DPN), one of the most common chronic complications of diabetes mellitus. *Fan et al.* developed engineered MSC-EVs *via* transfecting parent cells with miR-146a, and this modified EVs markedly inhibited the peripheral blood inflammatory monocytes and the activation of ECs *via* inhibiting Toll-like receptor (TLR)-4/NF-*κ*B signaling pathway [133]. Considering that both biochemical and electrical cues are essential for nerve regeneration, polypyrrole nanoparticles (PpyNps) with electrically conducting are widely exploited both *in vitro* and *vivo* for nerve regeneration. *Sing et al.* fused BMSC-EVs with PpyNps containing liposomes by means of freeze-thaw method, and this hybrid NVs coupled with exogenous electrical stimulation synergistically enhanced regenerative outcome, offering a new treatment approach in DPN [134].

## 9. Renal Injury

Acute kidney injury (AKI) is defined by many factors that contribute to an abrupt loss of kidney function including a rapid increase in serum creatinine, a decrease in urine production, or both. AKI is a common complication in hospitalized patients with high morbidity and mortality [135]. Moreover, AKI is associated with an increased risk of chronic kidney disease (CKD) and end-stage renal disease. However, there are no definitive therapeutic methods to cure established AKI or prevent it from progressing to CKD. Preclinical studies have demonstrated that multiple cell-derived such as MSCs [136, 137], endothelial colony forming cells [138], renal tubular epithelial cells [139], urinary-derived EVs [140] have good efficacy in the treatment of AKI. And several studies have shown that therapeutic cargos (including proteins, miRNAs, plasmids, and drugs) engineered EVs can enhance the therapeutic efficacy of AKI.

Klotho, a protein that has a protective effect in the kidney, has been reported to be able to treat AKI. Grange et al. loaded Klotho recombinant protein into urinary-derived EVs using the Exo-Fect transfection reagent. EVs loaded with Klotho proteins significantly improved the recovery of renal function in an acute tubular injury model [140] (Table 7). EVs derived from engineered MSCs overexpressing miR-let7c delivered exogenous miR-let7c alleviated renal fibrosis [141]. Furthermore, EVs encapsulating miR-29 administrated by intramuscular injection showed more accumulation in injured-renal, reduced kidney fibrosis in the CKD model and attenuated muscle wasting which is one of the complications of CKD more evidently [142]. The macrophage-derived microvesicles with inflammation tropism and anti-inflammation effects were applied for targeted delivery of DEX into inflamed kidney to alleviate inflammation and fibrosis [143]. Similarly, IL-10 was loaded into EVs from macrophage for AKI treatment and prevention of CKD considering that IL-10 with inflammatory effects however with several limitations includes instability and tendency to activate leukocytes in the circulation [144]. Moreover, studies have shown that melatonin stimulated MSC-derived EVs enhanced the protective effect of kidney injury in the CKD disease model [145]. In conclusion, these findings demonstrate the effectiveness and security of a novel therapeutic cargo delivery strategy with promising clinical applications. Besides, to increase EV stability and retention in the treatment of AKI, a class of RGD hydrogels recently was formulated based on that RGD peptide can bind to integrins which are presented on the surface of EVs. And RGD hydrogels showed extraordinary effects on promotion of proliferation, antifibrosis, antiapoptosis, and proautophagy [146].

## 10. Musculoskeletal Diseases

### 10.1. Osteoporosis

As one of the most common chronic, age-related disease, osteoporosis, characterized by low bone mass and deterioration in bone microarchitecture, is related to the imbalance of osteoblasts synthesizing bone and osteoclasts breaking down bone [147]. Accumulating evidences demonstrate that EVs could serve as a novel therapeutic tool or biomarker of osteoporosis. For example, as a potential biomarker of osteoporosis, has-circ-0006859 is upregulated in serum of osteoporosis compared health people with high sensitivity and specificity [148] and as a promising therapeutic agent, it has been widely reported that EVs derived from BMSCs can be used to alleviate osteoporosis. Furthermore, as a potential gene delivery tool, EVs from BMSCs packaging lncRNA MALAT 1[149], circRNA RTN4 [150], miR-935 [151], miR-29a [152], and miR-150-3p [153] exhibit stronger therapeutic efficacy compared with naive EVs. The bone-targeting ability of EVs is another aspect, which is associated with the source of EVs. For example, Song et al. found that EVs derived from EC showed distinct superiority in bone targeting ability in contrast with EVs derived from BMSCs and MC3T3[154]. Nevertheless, the specific targeting and therapeutic ability of natural EVs are limited. For example, Luo et al. found that bone marrow stromal cell- (ST-) derived EVs (ST-EVs) can strengthen osteoblastic differentiation of BMSCs in vitro. However, the ST-EVs failed to prevent osteoporosis in the osteoporotic mice model because a large number of ST-EVs accumulated in the lung and liver are not bone tissue, and this phenomenon evokes researchers to employ a type of aptamer which was displayed in the surface of EVs (apt-EVs) to target BMSCs of bone marrow and more importantly, this apt-EVs endowed EVs with same excellent therapeutic efficacy in vivo [155] (Table 8). It is critical to inhibit adipogenic differentiation and promote osteogenic differentiation from BMSCs for the therapy of bone loss-related disease. It has been reported that miR-188 was involved in switch between osteogenesis and adipogenesis, and antagomiR-188 can play a role in promoting bone formation [156, 157]. Hu et al. constructed a type of hybrid NVs *via* fusing EVs which display C-X-C motif chemokine receptor 4 (CXCR4) on the surface with liposomes carrying antagomiR-188. CXCR4^+^ EVs could be recruited selectively to the bone marrow which is enriched in stromal cell-derived factor 1 (SDF1), a ligand of CXCR4 and predominantly expressed by BMSCs. Thus, CXCR4 endowed hybrid NVs with targeting ability and antagomiR-188 enabled hybrid NVs to promote bone formation. Moreover, this kind of hybrid greatly strengthened cargo loading efficiency compared with separately using of EVs and greatly enhanced targeting capability in contrast with only application of liposomes [158].

### 10.2. Osteoarthritis

**Figure 3:**
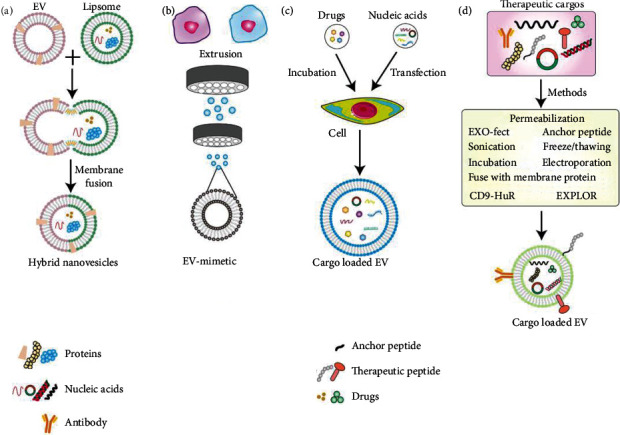
Approaches for loading therapeutic cargos into EVs. (a) Cargo encapsulated hybrid nanovesicles are generated via fusing EVs with therapeutic agents containing liposome. (b) EV mimetic packaging a large number of bioactive molecules is formulated by extrusion. (c) Therapeutic cargos primarily including drugs and nucleic acids are packaged into EVs through indirectly cellular transfection and incubation. (d) Therapeutic cargoes including nucleic acids, drugs, proteins, therapeutic peptides, and antibody are transferred into EVs by permeabilization, Exo-Fect, sonication, freeze/thaw and electroporation, CD9-HuR, EXPLOR, fuse therapeutic with EV membrane protein, and therapeutic peptide and antibody also can be linked to EV by anchor peptides.

Osteoarthritis (OA) is the most prevalent type of chronic degenerative joint disease that affects over 300 million people throughout the world [159, 160]. During the development of OA, the pathologic changes in joints may involve cartilage damage, the subchondral bone remodeling, inflammatory activation in the synovium, etc. [161]. The current treatment of OA mainly includes pain management and arthroplasty for end-stage disease [162]. However, these strategies fail to achieve satisfactory results in improving bone homeostasis and delaying OA progression. Recently, the role of EVs has increasingly attracted attention in the therapy of OA, and the studies mainly focus on exploring the diagnostic significance and biological effects of endogenous EVs during OA and therapeutic effects of EVs predominantly from MSCs such as BMSCs, ADSCs, synovial MSCs (SMSCs), and infrapatellar fat pad MSCs [161]. The function of EVs in diagnosis and therapy of OA are mainly ascribed to EV content miRNAs (including miR-6878-3p, miR-210-5p, miR-26a-5p, miR-146a-5p, miR-6821-5p, and miR-92a-3p), lncRNAs (including lncRNA PCGEM1, and lncRNAKLF3-AS1), and proteins like CD73/ecto-5′-nucleotidase [163–167]. In addition, in order to augment OA treatment efficacy, the modified EVs have also been proposed. The modification strategies mainly involve cargo loading, bone-targeting modification, and combined application with biomaterials. miR-210 modified BMSC-EVs exhibit superiority in anti-inflammation and antiapoptosis of chondrocytes compared with BMSC-EVs [168]. EVs derived from MSCs overexpressing miR-92a-3p are capable of strengthening cartilage proliferation and matrix gene expression [167]. It has been suggested that EVs derived from SMSCs played a crucial role in promoting chondrocyte proliferation and migration. Nevertheless, SMSC-EVs have the drawbacks in inhibiting the synthesis of extracellular matrix (ECM) protein including aggrecan and collagen II. To overcome these shortcomings, SMSC-140-5-EVs was fabricated *via* overexpressing miR-140-5 into SMSCs because miR-140-5 has a function in cartilage homeostasis and targeting RalA to enhance SOX9 and aggrecan expression in SMSCs [169]. Apart from loading cargos into EVs by transfecting gene into donor cells, the direct loading of cargos has also been employed (Figure 3). For example, activating transcription factor4, capable of modulating chondrocyte proliferation and bone formation, was loaded into serum EVs derived from OA mice *via* electroporation [170]. Furthermore, to overcome insufficient targeted delivery of EVs to chondrocytes across the dense, nonvascular ECM of cartilage, Liang et al. loaded miR-140 to DC-derived EVs which were engineered by chondrocyte affinity peptide (CAP) *via* fusing with the LAMP2B protein on the surface of EVs to realize targeted delivery of miR-140 to chondrocytes [171]. Transplantation of synovial fluid-derived MSCs (SF-MSCs) is critical for OA treatment but is less effective as a cartilage substitute owing to their fibroblastic capability after transplantation [172, 173]. Kartogenin (KGN) is a recently discovered small molecule compound that can mediate SF-MSC-specific differentiation into chondrocytes. However, KGN is characterized by low water solubility, which makes it difficult for accurate administration, easy to form precipitates in the cell, and exhibit low effective concentration thereby limited its chondrogesis-promoting activity. Xu et al. engineered EVs derived from DCs to achieve targeted delivery of KGN to SF-MSCs, even dispersion of KGN in the cytosol, effective concentration in the SF-MSCs, enhanced chondrogenesis of SF-MSCs by fusing E7 peptide capable of targeting SF-MSCs with EV protein LAMP2B (E7-EVs), and delivering KGN into EVs *via* electroporation [173]. In the treatment of OA, biomedical scaffolds have also been proposed. The combined application of HucMSC-EVs with acellular cartilage ECM scaffold exhibited better therapeutic effects compared with the single HucMSC-EV group. Specifically, in the EV-scaffold group, the deep layer cells were arranged in a typical vertical band which were similar to the cellular arrangement in natural cartilage, while the cells in the repaired tissue in the human MSC-EV group were arranged in a disorderly manner, which was significantly different from the normal cartilage structure [174]. Meanwhile, a kind of 3D printed scaffold with radially oriented channels and composed of decellularized cartilage ECM, and gelatin methacrylate (GelMA) hydrogel was loaded with EVs and then significantly facilitated the cartilage regeneration in the OA rabbit model [175].

### 10.3. Fracture

Fracture is a common traumatic injury, the bone itself possesses a certain ability to repair and the regenerative process comprises inflammation, angiogenesis, stem cell differentiation, osteogenesis, and chondrogenesis. However, approximately 5%-10% of fractures are complicated by delayed healing or nonunion [176, 177]. A large number of studies demonstrate that EVs could promote fracture recovery *via* mediating immunomodulation, osteogenesis, and angiogenesis. For example, BMSC-EVs can be encapsulated by ECs and osteoblast cell effectively and accelerate osteogenesis and angiogenesis *via* BMP-2/Smad1/RUNX2 and HIF-1*α*/VEGF signaling pathway respectively [177]. Furthermore, MSC-EVs under hypoxia were able to strengthen angiogenesis, proliferation, and migration to a greater extent compared with EVs cultured in normal condition *via* transferring miR-126 [178]. In recent years, “osteoimmunology” was introduced into bone regeneration, suggesting strong crosstalk between immunology and the skeletal system [179]. For example, M2 macrophage-derived EV miR-5106 can be transferred into BMSCs and induced BMSC osteoblastic differentiation *via* targeting SIK2 and SIK3 genes [180]. Large segmental bone defect repair based on EVs need to meet the requirements of promoting bone cell proliferation, the reconstruction of internal vasculature, and topical delivery and controllable release of functional EVs at the defect site. Thus, a type of EV-mediated bone scaffold system was constructed. In this system, EVs from ATDC5 overexpressing VEGF were loaded into a class of microscale porous PCL scaffold *via* a CD63-specific EV anchor peptide CP05 (PCL-CP05-EV-VEGF). As a result, PCL-CP05-EV-VEGF was capable of promoting the ingrowth of new tissues, provided a 3D space for vasculature remodeling and better promoted bone regeneration [181–183]. In combined application of modified EVs with biomaterials, EMs were constructed from the noggin-knockdown hMSCs cultured in conditioned osteogenic medium (EMs-OMN) *via* extrusion. The production of this EMs was more scalable compared with EVs derived from hMSCs, and EM-OMN exhibited robust bone regeneration because the suppressed expression of noggin enhanced osteogenic properties of EMs-OMN. More importantly, a further enhanced osteogenesis *in vitro* and *in vivo* was observed in the EM-OMN laden MeGC hydrogel [184]. Osteogenic EVs were loaded into 3D Ti-scaffolds with multiple advantageous properties like biocompatibility, nontoxicity, good mechanical strength, optimal porosity for cell migration and proliferation, and high surface areas for cell attachment. The EV-coated Ti-scaffolds showed more excellent bone regeneration in contrast with EV-free Ti-scaffold implants [185]. Similarly, the titanium nanotubes functionalized EVs from the BMP2-stimulated macrophages promoted osteogenic differentiation and can avoid ectopic bone formation and reduce adverse effects [186]. It is considered that a suitable bone biomaterial should possess ability both in mediating osteogenesis and manipulating the immune response, thereby exerting a synergistic effect for achieving satisfactory osseointegration [187]. For instance, the implantation of synthetic biomaterials may contribute to an activated M1 phenotype that subsequently secretes multiple proinflammatory cytokines, and the long-term exposure to inflammatory cytokines may eventually lead to osseointegration failure [188, 189]. Conversely, the M2 phenotype macrophages possess capability in secreting anti-inflammatory cytokines, which is favorable for bone regeneration environment formation [188, 190]. BMSC-derived EVs with osteogenic differentiation and immunomodulatory advantages were incorporated on tannic acid (TA) modified sulfonated polyetheretherketone (SPEEK) which can ensure sustained release of EVs and was in favor of improving osseointegration. EV-loaded TA-SPEEK enabled macrophage M2 polarization (an anti-inflammatory phenotype) *via* the NF-*κ*B pathway, which represented more favorable bone immune microenvironment that was beneficial for further BMSCs osteogenic differentiation [190].

### 10.4. DMD

Duchenne muscular dystrophy (DMD) is a life-threatening disorder that is caused by the absence of functional dystrophin protein, resulting cell membrane fragility, muscle damage, inflammation, fibrosis, and ultimate degeneration [191, 192]. EVs from cardiosphere-derived cells (CDCs) were reported to be a therapeutic candidate for DMD; notably, the obtaining of CDCs is time-consuming and highly technical for isolation and purification maintenance [193]. Systemic administration of EVs derived from hMSCs, murine serum, and myotubes can delay pathological progression *via* improving membrane integrity in mdx mice without detectable toxicity [193]. However, these approaches were unable to achieve cure effects for DMD. Recently, in order to further augment therapeutic efficiency of EVs for DMD, several engineered EVs have been proposed. Inflammation was considered a major target for DMD therapy, and corticosteroids with capability of decreasing inflammation are regularly used in clinic, but the use of corticosteroids is accompanied with a large number of side effects. IL-6, a key inflammatory cytokine, plays a vital role in skeletal muscle pathophysiology through two different mechanisms: the classical pathways participating in anti-inflammatory and transsignaling pathways conversely, mediating chronic inflammation. In light of this, EVs derived from BMSCs were designed to express IL6 signal transducer decoy receptors to selectively inhibit the IL6 transsignaling pathway and have no effects on classical signaling, thereby provided a potential for the treatment of DMD [194]. More importantly, the importance of the IL6 transsignaling pathway in muscle-related pathologies was first elucidated, and decoy receptor EV platform may combine multiple bioactive molecules or targeting ligands to further enhance various tissue therapeutic efficacy. Myostatin propeptitde can play a role in inhibiting mature myostatin. However, the direct administration of myostatin propeptitde was restricted for broad application because of safety problem, poor serum stability, and low delivery efficiency. Ran et al. constructed a delivery platform *via* fusing the inhibitory domain of myostatin propeptide with EV membrane protein CD63, which increased delivery and serum stability of propeptide and enhanced the inhibitory efficacy of myostatin propeptide. As a result, strengthened muscle mass and functional protection without detectable toxicity in mdx mice were achieved [192].

## 11. Skin Wound

Delayed wound healing and scar formation remains two main challenges in skin wound defects. It has been widely reported that EVs from stem cells such as HucMSCs and menstrual blood-derived MSCs can participate in skin wound healing [195, 196]. The modification of EVs in the therapy of skin wound mainly considers encapsulating cargos into EVs and the combined application of EVs with nanomaterials. As for therapeutic gene delivery, EVs secreted from BMSCs overexpressing TSG-6 significantly enhanced anti-inflammation and alleviated the formation of pathological scar injury [197]. ADSC-EVs greatly enhanced granulation tissue formation and angiogenesis and obviously promoted wound healing [198]. Moreover, direct engineering of EVs is also employed to repair wound defects. For example, miR-21-5p mimics, a novel therapeutic agent for diabetic wound recovery, was transferred into EVs derived from ADSCs by electroporation, and the engineered EVs exhibited excellent therapeutic outcomes in mediating proliferation and migration of keratinocytes through Wnt/*β*-catenin signaling pathway *in vitro* and promoted reepithelization, collagen remodeling, and angiogenesis in diabetic wound models [199] (Table 9). Compared with transfecting cargos into donor cells, electroporation technique may possess the advantages of better encapsulation efficacy, and the process is quick. However, admittedly, it is limited to extensive application because this method can undermine the integrity of EVs thereby influenced the efficiency of wound recovery [200–202].

**Figure 4:**
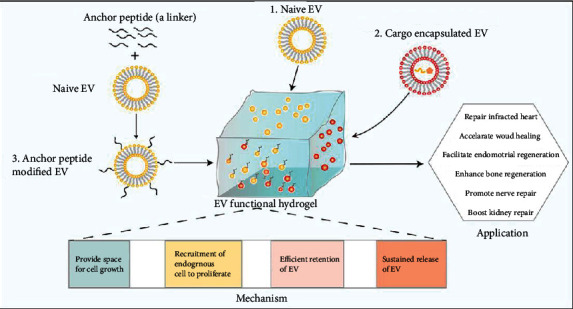
The role of EV functioned hydrogel. Naive EVs and cargos encapsulated EVs can be combined with hydrogels. In addition, in order to increase the retention efficiency of EVs in hydrogel, anchor peptide can serve as a linker to bridge EV with hydrogel. EV functioned hydrogel is capable of promoting infarcted heart repair, accelerating wound healing, boosting kidney repair, strengthening nerve reparative outcome, and enhancing bone and endometrial regeneration.

Emerging studies have been reported to combine EVs with nanomaterials such as hydrogel (Figure 4), metal nanoparticles, and other kinds of dressings to provide a better solution for synergistically enhancing skin tissue regeneration. It is believed that self-healing hydrogels are the most promising wound dressings because they possess the peculiarity of hemostatic ability, self-healing, controlled biodegradation, being injectable, tissue-adhesion, antibacterial activity, anti-ultraviolet, sequential bioactive molecule release, and excellent biocompatibility [203–206]. For example, Xu et al. fabricated a thermosensitive polysaccharide-based FEP hydrogel scaffold with antibacterial activity, fast hemostatic ability, good UV-shielding, and pH-responsive EV release performance. And eventually, this kind of hydrogel enhanced its high ability of promoting diabetic wound healing with less scar formation and skin appendage regeneration [207]. Recently, an extremely effective three-dimensional porous natural-based methyl-cellulose-chitosan hydrogel was proposed to load placental MSC-derived EVs to synergistically promote severe wound healing [204]. Xu et al. found that chitosan/silk hydrogel sponge loaded with platelet-rich plasma EVs was more successful in accelerating wound healing compared with single use of EVs or hydrogel [207]. It is also reported that HUVEC-derived EVs combined with GelMA hydrogel might provide a potential prospect for accelerated cutaneous wound healing [208]. Apart from hydrogels, other kinds of biomaterials are also used to combine with EVs. An antioxidant wound dressing OxOBand composed of polyurethane was synthesized and supplemented with EVs secreted from ADSCs, which can effectively alleviate hypoxia and oxidative stress, induce angiogenesis, and exhibit faster wound closure [209]. Zhang et al. have suggested that marine sponge Haliclonasp spicules, as a novel microneedle, could provide a safe and effective tool to deliver EVs to the deep layer of skin thus increased the skin absorption of EVs and eventually could produce significant therapeutic effects against skin photoaging in mice [210]. Chitosan-silk fibroin dressing loaded with silver nanoparticles with broad-spectrum antimicrobial ability and EVs from HucMSCs (CTS-SF/SA/Ag-EVs dressing) was able to effectively inhibit the growth of bacterial and enhance wound healing in an infected diabetic wound model [211]. Metal oxide nanomaterial like Fe_3_O_4_ with low toxicity, advanced targeting capability, biodegradability, high saturation magnetization, and good biocompatibility was employed to increase the accumulation of EVs s at injury site [212, 213]. Recently, Li et al. have successfully constructed IONP-labeled EVs derived from MSCs and found that IONP can serve as a magnet-guided navigation tool, increased the EV accumulation at the cutaneous wound thereby augmented wound healing, reduced scar formation, and increased collagen expression [214].

## 12. Other Diseases

Besides the disease mentioned above, modified EVs are also involved in other diseases. The fetal inflammatory response is associated with neonatal mortality and morbidity, which often results in spontaneous preterm birth (PTB). Accumulating studies are testing to inhibit inflammation through suppressing the inflammatory transcription factor NF-*κ*B pathway; however, they are limited to clinical application partly due to key pharmacological issues such as placental permeability and low efficiency of drug delivery. In light of these, Sheller-Miller et al. and Yim et al. engineered EVs that contained an inhibitor of NF-*κ*B called superrepressor (srI*κ*B) using an innovative tool named “EV for protein loading *via* optically reversible protein-protein interactions” (EXPLORs) which enabled efficient delivery of protein cargos into the cytosol of target cells through controllable, reversible detachment from the EVs, thus allowing for decreased NF-*κ*B activation and the fetal inflammatory response and delayed LPS-induced PTB [219, 220]. Moreover, EXPLORs implemented for srI*κ*B loading into EVs were also used to alleviate systemic inflammation in sepsis [221] (Table 10). To improve endometrial regeneration and fertility restoration, an injectable ADSC-EV laden Ag-S coordinated PEG hydrogel was generated, and EV-hydrogel exerted effects on sustained release of EVs, antibacterial activity, promotion of neovascularization, suppression of fibrosis, and increased endometrial receptivity [222]. Triiodothyronine (T3) at low concentration can mediate the oligodendrocyte progenitor cell differentiation and may promote myelin regeneration. However, systematically administrated T3 at low concentration failed to reach the injured area and high dosage of T3 resulted in various side effects like peripheral immune reaction and cytotoxicity. Given the advantages of EVs on drug delivery, Xiao et al. loaded T3 into EVs from neural stem cells overexpressing ligand PDGF-A (PDGF-A-EV+ T3), which can target to the lesion of the spinal cord because PDGFR is significantly elevated in demyelinated areas. More importantly, targeted PDGF-A-EV loaded with low dosage of T3 remarkably enhanced the delivery of T3 and significantly delayed experimental autoimmune encephalomyelitis development [223].

## 13. Conclusions and Future Directions

EVs have shown great potential in multiple tissue and organ (e.g., lung, neuro, brain, skin, diabetes, and eye) regeneration, and clinical trials with allogenic and autologous stem cell derived EVs are underway. Furthermore, modified EVs greatly improved therapeutic outcome. In this review, we summarized various EV engineering strategies in diverse tissue and organs and described their specific characteristics. As cargo carrier, EVs show many advantages over several synthetic nanoparticles such as avoidance of phagocytosis by macrophages because of the existence of “do-not-eat-me” signal CD47 on EV surface, low toxicity, and immunogenicity. In addition, the biodistribution of EVs at injured sites tends to be the premise of successful repairs; thus, diverse approaches involving targeted peptide, several proteins, antibody, magnetic nanoparticles, and aptamer are used to strengthen tissue and organ targeting capacity. EMs produced by extrusion, self-assembly EV mimicking nanocomplex, and edible food derived vesicle-like nanoparticles with biocompatibility and stability are more scalable in large production and more efficient in encapsulating therapeutic agents. Considering the retention and therapeutic efficacy of EVs are transient, the bioactive materials like hydrogels, scaffolds, and dressings are promising strategies for precise and sustained release of EVs.

Despite the promising prospect of modified EVs for clinical application, there are still several major challenges needed to be addressed. For EV manufacturing, selecting parent cells and culture condition are two essential aspects which are associated with EV composition, bioactivity, homing property, and production. In addition, understanding the EV comprehensive physicochemical characterization is necessary for EV engineering and safety in clinical application; however, the characterization of EVs remains challenging because of the inherent heterogeneity. In addition, EV isolation methods may affect EV purity, quantity, specificity, and exosomal membrane integrity, which have effects on engineering EV. For cargo loading, the loading efficiency, drug property, and experimental installation, complexity of loading process, and the effects on EV properties are needed to be considered. The targeting modification of EVs may affect the EV membrane protein, cause immune response, and affect the property of recipient cells. Although aptamers are considered as a promising method to achieve targeting ability, high cost and insufficient studies of binding sites restrict further application. For the combined application of EVs biomaterials like hydrogel, dressing, and scaffolds, the biomaterials possess different properties, and the administration mode is also different; thus, it is significant to choose a suitable one for a specific disease, and it is essential to take the properties of biomaterials into consideration including the possibility in leading to immune response. Furthermore, keeping sequential release of EVs is one of the important function of biomaterials, and understanding the dynamic release of EVs *in vivo* and determining the quantity of EVs loaded into biomaterials may achieve better therapeutic efficacy. In addition, owing to the pathological characteristics of diseases and tissue are different, we believe that developing and choosing an appropriate EV engineering strategy for specific disease therapy is promising for precise medicine. Besides, it would be possible to extend a certain engineering strategy for more extraordinary therapeutic efficacy in multiple diseases, and the coordination of two or more engineering strategies may enable a construction of multifunctional platform which is more potent in tissue and organ regeneration. Notably, the synthesis of platform tends to be more intricated.

In conclusion, modified EVs can serve as a promising candidate for tissue and organ regeneration. Although there are key issues needed to be addressed for modified EV clinical transition, we believe that the difficulties will gradually be solved with the medical research development, and we anticipate that this review will provide new possibilities for better engineering EVs.

## Figures and Tables

**Table 1:** Modified EVs in the therapy of neurological disorders.

Diseases	EV Sources	Specific substrates	Modification methods	Modified EVs	Biological effects	Ref.
SCI	BMSC	miR-26a	Cell transfection	miR-26a-EV	Axonal regeneration and less glial scarring	[24]
SCI	BMSC	miR-124-3p	Cell transfection	miR-124-3p-EV	M2 polarization; antiapoptosis	[23]
SCI	ADSC	lncGm37494	Cell transfection	lncGm37494-EV	M2 polarization	[25]
SCI	BMSC	GIT1	Cell transfection	GIT1-EV	Less glial scar formation; anti-inflammation and apoptosis	[26]
SCI	BMSC	Sonic hedgehog	Cell transfection	Shh-EV	Neuronal regeneration	[27]
SCI	BMSC	Fe_3_O_4_-treated BMSCs	Magnetic particles; extrusion	Mag-EMs	EMs accumulation at injured site; more therapeutic cargos packaging	[28]
SCI	UCB-MSC	Macrophage membrane-fused MSCs	Extrusion	MF-EMs	Targeting ability; anti-inflammation	[29]
SCI	hMSC	Peptide-modified hydrogel	Biomaterials	pGel-EV	Efficient retention and sustained release of EVs; nerve recovery	[30]
SCI	M2 macrophage	Berberine	Ultrasonic	Ber-EV	Targeted delivery; anti-inflammatory and antiapoptotic effect	[10]
SCI	HucMSC	PTX; BSP; linearly ordered collagen scaffolds	Incubation; extrusion anchor peptide; biomaterials	LOCS-BSP-PTX-EMs (LBMP)	High retention of EMs-PTX within scaffolds; neuron formation	[21]
Stroke	BMSC	miR-17-92	Cell transfection	miR-17-92þ-EV	Axon-myelin remodeling and electrophysiological recovery	[34]
Stroke	IPAS	circSHOC2	Cell transfection	circSHOC2-IPAS-EV	Antiapoptosis and less neuronal damage	[35]
Stroke	HEK293	NGF; RVG peptide	Cell transfection; fuse targeted peptide with LAMP2B	NGF-RVG-EV	Targeted delivery; anti-inflammation; cell survival	[36]
Stroke	BMSC	c(RGDyK) peptide; cholesterol-modified miR-210	Click chemistry; incubation	MiR-210-RGD-EV	Targeted delivery; angiogenesis	[39]
Stroke	BMSC	Fe_3_O_4_-harboring BMSCs	Magnetic nanoparticles; serial extrusion	Mag-EMs	Targeted delivery	[40]
Stroke	Macrophage	Edaravone	Incubation	Edv-EV	Improvement of Edv bioavailability and brain targeting; neuroprotection effect	[11]
PD	Murine DC	shRNA minicircles; RVG peptide	Electroporation; fuse RVG with LAMP2B	shRNA-MC-RVG-EV	Targeted delivery; less alpha-synuclein aggregation	[43]
PD	HEK293T	DNA aptamers; RVG peptide	Aptamer; fuse targeted peptide with LAMP2B	Apt-RVG-EV	Targeted delivery; less *α*-synuclein aggregates and motor impairments	[46]
PD	imDC	RVG peptide; curcumin; ANP; siSNCA	Self-assembly EV-like nanocomplex	C/ANP/S-REV	Targeted delivery; nanoscavenger” for clearing *α*-synuclein and less immune activation	[50]
PD	Serum	Dopamine	Incubation	DA-EV	More brain distribution of dopamine effects	[49]
PD	Mononuclear phagocyte	Catalase	Incubation; permeabilization; freeze-thaw cycles; sonication; extrusion	CAT-EV	Anti-inflammation	[48]
AD	HEK-293T and BMSC	miR-29b	Cell transfection	miR-29b-EV	Less the pathological effects of amyloid-*β* (A*β*) peptide	[52]
AD	Plasma	Quercetin	Ultrasound incubation	Que-EV	Improvement of brain targeting and Que bioavailability	[55]
AD	Macrophage	Curcumin	Pretreated donor cells	Cur-EV	Improved bioavailability of cur; less phosphorylation of the tau protein	[51]

**Table 2:** Modified EVs in the therapy eye-related diseases.

Diseases	EV Sources	Specific substrates	Modification methods	Modified EVs	Biological effects	Ref.
Pathological retinal angiogenesis	EC	Anchoring peptide CP05; KV11 peptide	Link therapeutic peptide to EV via anchoring peptide	KV11-CP05-EV	Less neovascularization and vessel leakage	[62]
CNV	Treg	VEGF antibody	Conjugate antibody to EV via a peptide linker (cL)	CL-aV-EV	Inhibition of inflammation and VEGF activity	[63]

**Table 3:** Modified EVs in the therapy of lung diseases.

Diseases	EV Sources	Specific substrates	Modification methods	Modified EVs	Biological effects	Ref.
ALI	BMSC	miR-30b-3p	Cell transfection	miR-30b-3p-EV	Anti-inflammation more effectively	[66]
ALI	Platelet	DEX	Incubation	DEX-EV	Targeted delivery and less side effects	[68]
ALI	HEK293 cells	Curcumin; RBP	Incubation; fuse targeted peptides (RBP) with LAMP2B	RBP-EV	Targeted delivery and increased therapeutic effect	[69]
COVID-19	Ginger derived lipid vesicles	miR396a-5p or rlcv-miRrL1-28-3p	Incubation	miRNA-EV	Remarkably suppressed inflammation and cytopathic effect	[73]
COVID-19	HEK-293T cells	SiRNA-GFP; RBD	Electroporation; cell transfection with RBD-VSVG fusion vector	SiRNA-GFP-RBD-EV	Targeted delivery	[74]
COVID-19	HEK-293T cells	PM targeting sequence	Fuse PM targeting sequence with ACE2	PM-ACE2-EVs	Increase the accumulation of ACE2 on EV and block the interaction of virus with cells	[75]
IPA	Fibroblast cell	CLD-loaded liposomes	Hybrid nanovesicles	CLD-hybrid nanovesicles	Targeted delivery of antifibrotic drug with high efficiency	[82]
AA	M2 macrophage	Dnmt3aos^smart silencer^; PLGA nanoparticles	Synthetic EV-like nanocomplex	EV membrane coated Dnmt3aos^smart silencer^	Increase nanocomplex stability; targeted delivery of nucleic acid	[86]

**Table 4:** Modified EVs in the therapy of cardiovascular related diseases.

Diseases	EV Sources	Specific substrates	Modification methods	Modified EVs	Biological effects	Ref.
MI	hMSC	lncRNA KLF3-AS1	Cell transfection	KLF3-AS1-EV	Antiapoptosis and pyroptosis	[89]
MI	BMSC	miR-210	Cell transfection	miR210-EV	Antiapoptosis effects	[90]
MI	BMSC	GATA-4	Cell transfection	GATA-4-EV	Antiapoptosis and promotion of cardiac function recovery	[99]
MI	CDC	CMP	Fuse targeted peptide with LAMP2B	CMP-EV	Higher cardiac EV retention; less apoptosis	[101]
MI	BMSC	Fe_3_O_4_-treated BMSCs	Magnetic particles; Extrusion	Mag-EM	Retention of EMs within infarcted heart; cardiac function recovery	[105]
MI	Rat serum	GMNP_EC_ with anti-CD63 and anti-MLC antibody	Magnetic nanoparticles; antibody	GMNP_EC_–EV	Recruitment, transport, and targeted delivery of EVs; angiogenesis	[106]
MI	iCM	Hydrogel patch	Biomaterials	Patch-EV	Sustained delivery of EVs	[108]
AS	M2 Macrophage	HAL	Electroporation	HAL-EV	Excellent inflammation-tropism and anti-inflammation effects	[111]
AS	HucMSC	miR-145	Cell transfection	miR-145-EV	Inhibition of cell migration and atherosclerotic plaque	[110]
AS	HucMSC	HucMSC	Improved extrusion methods	FT/EM	Higher production yield of FT/EM; anti-inflammation	[112]

**Table 5:** Modified EVs in the therapy of digestive diseases.

Diseases	EV Sources	Specific substrates	Modification methods	Modified EVs	Biological effects	Ref.
Liver fibrosis	ADSC	miR-122	Cell transfection	miR-122-EV	Inhibition of liver fibrosis	[113]
Liver fibrosis	Fibroblast-like MSC	SiRNA or ASO targeting STAT3	Electroporation	SiRNA-EV or ASO-EV	Inhibition of STAT3 expression and ECM deposition	[115]
Liver injury	HEK293T	HuR; antimiR-155 fused with AREs	CD9-HuR fusion protein system	CD9-HuR+antimiR-155-AREs-EV	High encapsulation of antimiR-155; antifibrosis	[116]
IBD	HucMSC	miR-326	Exo-Fect™ agent	miR-326-EV	anti-inflammation effects	[120]
IBD	BMSC	HO-1	Cell transfection	HBM-EV	Anti-inflammation effects	[122]
IBD	Ginger plant	SiRNA-CD98; ginger-derived lipid vehicles	Turbo Fect reagent	GDLV-CD98	Targeted delivery; lower expression of CD9	[72]

**Table 6:** Modified EVs in the therapy of diabetes and its complication.

Diseases	EV Sources	Specific substrates	Modification methods	Modified EVs	Biological effects	Ref.
Diabetes	BMDM	miR-690	Cell transfection	miR-690-EV	Glucose tolerance and insulin sensitivity	[126]
Diabetes	Serum	BAY55-9837 peptide; SPIONs	Electroporation; magnetic particles	SPION-BAY-EV	Targeted delivery of BAY55-9837; increase insulin secretion	[127]
DN	BMSC	miR-let-7a	Cell transfection	miR-let-7a-EV	Antiapoptosis	[130]
DPN	BMSC	miR-146a	Cell transfection	miR-146a-EV	Reduce neurovascular damage	[133]
DPN	BMSC	PpyNps containing liposomes	Hybrid nanovesicles	PpyNps-hybrid nanovesicles	Provide electrical cues; Synergetic regenerative effect	[134]

**Table 7:** Modified EVs in the therapy of renal injury.

Diseases	EV Sources	Specific substrates	Modification methods	Modified EVs	Biological effects	Ref.
AKI	Fibroblast	Klotho	Exo-Fect agent	Klotho-EV	Renoprotective effects	[140]
AKI	PL-MSC	RGD hydrogels	Biomaterials	RGD hydrogel-EV	Augment retention and stability of EVs and treatment efficacy	[146]
Renal fibrosis	BMSC	miR-let7c	Cell transfection	miR-let7c-EV	Antifibrosis	[141]
CKD	Satellite cell	miR-29	Cell transfection	miR-29-EV	Antifibrosis	[142]

**Table 8:** Modified EVs in the therapy of musculoskeletal diseases.

Diseases	EV Sources	Specific substrates	Modification methods	Modified EVs	Biological effects	Ref.
Osteoporosis	BMSC	miR-935	Cell transfection	miR-935-EV	Osteoblast proliferation and differentiation	[151]
Osteoporosis	BMSC	miR-29a	Cell transfection	miR-29a-EV	Robust ability in angiogenesis and osteogenesis	[152]
Osteoporosis	ST	DNA aptamers targeting bone	Aptamer	Apt-ST-EV	Targeting delivery; osteoblastic differentiation	[155]
Osteoporosis	NIH-3T3 cell	CXCR4^+^ EV and liposomes carrying antagomir-188	Hybrid nanovesicles	Antagomir-188-hybrid NV	Increase bone-targeting; alleviate bone loss	[158]
Osteoporosis	BMSC	circRNA Rtn4	Cell transfection	Rtn4-EV	Reduced cytotoxicity and apoptosis	[150]
OA	BMSC	miR-210	Cell transfection	miR-210-EV	Proliferation and antiapoptosis	[168]
OA	BMSC	miR-92a-3p	Cell transfection	miR-92a-3p-EV	Promote cartilage proliferation	[167]
OA	SMSC	miR-140-5p	Cell transfection	SMSC-140-EV	Enhance cell proliferation and migration without damaging ECM secretion	[169]
OA	Serum	ATF4	Electroporation	ATF4-EV	Inhibit chondrocyte apoptosis	[170]
OA	DC	MiR-140; CAP peptide	Electroporation; fuse targeted peptide with LAMP2B	miR-140-CAP-EV	Deliver miR-140 to deep cartilage regions and promote bone regeneration	[171]
OA	DC	KGN; E7 peptide	Electroporation; fuse targeted peptide with LAMP2B	KNG-E7-EV	Bone-targeting capability and higher cartilage differentiation	[173]
OA	HucMSC	ACECM scaffolds	Biomaterials	ACECM-EV	Sustained release of EVs; osteochondral regeneration	[174]
Fracture	ATDC5	VEGF; PCL-scaffolds; anchor peptide CP05	Cell transfection; anchor peptide; biomaterials	PCL-CP05-VEGF-EV	High grafting efficiency of EVs; osteogenic differentiation; angiogenesis	[183]
Fracture	hMSC	3D Ti-scaffolds	Biomaterials	Ti-scaffold-EV	Bone regeneration ability	[185]
Fracture	BMP2-treated macrophage	Titanium oxide nanotubes	Biomaterials	NT-BMP2-EV	Avoid ectopic bone formation; osteogenic differentiation	[186]
Fracture	BMSC	TA modified 3D porous SPEEK	Biomaterials	TA-SPEE-EV	Osteoimmunomodulation effect; sustained release of EVs; osseointegration	[190]
Fracture	hMSC	Noggin-suppressed hMSCs; MeGC hydrogel	Cell transfection; extrusion; biomaterials	H-OMN -EM	High yields of EM; robust bone regeneration	[184]
DMD	BMSC	IL6ST decoy receptors	Fuse interleukin receptor with EV protein	IL6ST-EV	Block IL6 transsignaling pathway	[194]
DMD	NIH3T3 cell	Myostatin propeptide	Fuse therapeutic peptide with CD63	Myostatin-EV	Increase delivery and propeptide stability	[192]

**Table 9:** Modified EVs in the therapy of skin wound.

Diseases	EV Sources	Specific substrates	Modification methods	Modified EV	Biological effects	Ref.
Pathological scar	BMSC	TSG-6	Cell transfection	TSG-6-EV	Further ameliorated pathological scar	[197]
DFU	ADSC	Nrf2	Cell transfection	Nrf2-EV	Enhance wound healing	[198]
DFU	ADSC	miR-21-5p	Electroporation	miR-21-5p-EV	Accelerate diabetic wound healing	[199]
DFU	SMSC	miR-126-3p; CS hydrogel	Cell transfection; biomaterials	CS-SMC-126- EV	Angiogenesis	[215]
DFU	ADSC	FHE hydrogel	Biomaterials	EV	Sustained release of EV; facilitate wound healing	[216]
DFU	Platelet-rich plasma	ZWP, chitosan/silk hydrogel	Biomaterials	PRP-ZWP/EV	Accelerate wound healing	[207]
DFU	ADSC	OxOBand dressing	Biomaterials	PUAO-CPO-EV	Less oxidative stress; anti-infection	[209]
DFU	ADSC	FEP hydrogel	Biomaterials	FEP-EV	PH-responsive EV release; fasten wound healing	[206]
Full-thickness skin defects	MEL-5 cell	PD-L1; PF-127 hydrogel	Cell transfection Biomaterials	PF-127-PD-L1-EV	Fasten reepithelialization	[217]
Full-thickness skin defects	ADSC	Alginate-based hydrogel	Biomaterials	Alg-EV	Improve wound closure	[218]
Full-thickness skin defects	HUVEC	GelMA hydrogel	Biomaterials	GelMA-EV	Accelerate wound healing	[208]
Full-thickness skin defects	HucMSC	Silver nanoparticle; CTS-SF/SA	Metal nanoparticle; biomaterials	CTS-SF/SA/Ag-EV	Broad-spectrum antimicrobial activity; accelerate wound healing	[184]
Photoaging	HucMSC	Marine sponge Haliclona sp	Biomaterials	SHS-EV	Increased the skin absorption of Exo; significant antiphotoaging effects	[211]
Burn injury	ADSC	Fe_3_O_4_ nanoparticles	Incubate magnetic particles with parent cells	Fe_3_O_4_-EV	Enhanced wound healing in a magnetic targeting way	[214]

**Table 10:** Modified EVs in the therapy of other disease.

Diseases	EV Sources	Specific substrates	Modification methods	Modified EVs	Biological effects	Ref.
PTB	HEK293T	SrI*κ*B	EXPLOR system	SrI*κ*B-EV	High loading efficiency of cargos postpone infection-induced PTB	[219]
Endometrial damage	ADSC	PEG hydrogel	Biomaterials	Hydrogel-EV	Angiogenesis anti-infective and antifibrotic effect	[222]
Sepsis	/	SrI*κ*B	EXPLOR system	SrI*κ*B-EV	Efficient encapsulation of cargos; anti-inflammation	[221]

## Abbreviations

EVs: Extracellular vesicles
EMs: Extracellular vesicle mimetics
MSCs: Mesenchymal stem cells
MVs: Microvesicles
MVBs: Multivesicular bodies
ILVs: Intraluminal vesicles
BBB: Blood-brain barrier
SPION: Superparamagnetic iron oxide nanoparticles
NP: Nanoparticle
SCI: Spinal cord injury
IONP: Iron oxide nanoparticle
hMSCs: Human MSCs
NVs: Nanovesicles
Edv: Edaravone
*α*-syn: Alpha-synuclein
PD: Parkinson's disease
shRNA-MCs: shRNA minicircles
BMSCs: Bone marrow MSCs
ASO: Antisense oligonucleotides
Shh: Sonic hedgehog
Mag: Magnetic particles
MF: Macrophage membrane-fused
pGel: Peptide-modified hydrogel
Ber: Berberine
PTX: Paclitaxel
BSP: Biospecificity peptide
LOCS: Linearly ordered collagen scaffolds
IPAS: Ischemic-preconditioned astrocyte
RVG: Rabies viral glycoprotein
RGD: Arg-Gly-AspD-Tyr-Lys
Apt: Aptamer
DA: Dopamine
CAT: Catalase
Que: Quercetin
Cur: Curcumin
AD: Alzheimer's disease
DC: Dendritic cell
PDR: Proliferative diabetic retinopathy
EndoMT: Endothelial-to-mesenchymal transition
EMT: Epithelial-mesenchymal transition
HucMSCs: Human umbilical cord MSCs
Treg: Regulatory T cells
CNV: Choroidal neovascularization
ROP: Retinopathy of prematurity
ADSCs: Adipose-derived MSCs
ALI: Acute lung injury
RBP: RAGE-binding peptide
DEX: Dexamethasone
PEVs: Platelet-derived extracellular vesicles
RBD: Receptor-binding domain
PM: Plasma membrane
IPF: Idiopathic lung fibrosis
CLD: Clodronate
NIN: Nintedanib
IPF: Idiopathic lung fibrosis
AA: Allergic asthma
PLGA: Polylactic-coglycolic acid
MI: Myocardial infarction
iPSCs: Induced pluripotent stem cells
CTP: Cardiac-targeting peptide
MSN: Mesoporous silica NP
HAL: Hexyl 5-aminolevulinate hydrochloride
FT: Freeze and thaw
TFF: Tangential flow filtration
HuR: Human antigen R
AREs: AU rich elements
RNAi: RNA interference
GDLVs: Ginger-derived lipid vehicles
HO-1: Heme oxygen-1
DM: Diabetes mellitus
DN: Diabetic nephropathy
DPN: Diabetic peripheral neuropathy
PpyNps: Polypyrrole nanoparticles
AKI: Acute kidney injury
CKD: Chronic kidney disease
ST: Bone marrow stromal cell
CXCR4: C-X-C motif chemokine receptor 4
SDF1: Stromal cell-derived factor 1
OA: Osteoarthritis
SMSCs: Synovial MSCs
ECM: Extracellular matrix
CAP: Chondrocyte affinity peptide
SF-MSCs: Synovial fluid-derived MSCs
KGN: Kartogenin
GelMA: Gelatin methacrylate
SPEEK: Polyetheretherketone
DMD: Duchenne muscular dystrophy
CDCs: Cardiosphere-derived cells
PTB: Spontaneous preterm birth
T3: Triiodothyronine.

## References

[1] Liu C., Su C. (2019). Design strategies and application progress of therapeutic exosomes. *Theranostics*.

[2] Zhang X., Zhang H., Gu J. (2021). Engineered extracellular vesicles for cancer therapy. *Advanced Materials*.

[3] Roefs M. T., Sluijter J. P. G., Vader P. (2020). Extracellular vesicle-associated proteins in tissue repair. *Trends in Cell Biology*.

[4] Huang J., Xiong J., Yang L., Zhang J., Sun S., Liang Y. (2021). Cell-free exosome-laden scaffolds for tissue repair. *Nanoscale*.

[5] An Y., Lin S., Tan X. (2021). Exosomes from adipose-derived stem cells and application to skin wound healing. *Cell Proliferation*.

[6] Lan Y., Xie H., Jin Q. (2022). Extracellular vesicles derived from neural EGFL-Like 1-modified mesenchymal stem cells improve acellular bone regeneration via the miR-25-5p-SMAD2 signaling axis. *Bioactive Materials*.

[7] Chen J., Ren S., Duscher D. (2019). Exosomes from human adipose-derived stem cells promote sciatic nerve regeneration via optimizing Schwann cell function. *Journal of Cellular Physiology*.

[8] Hazrati A., Malekpour K., Soudi S., Hashemi S. M. (2022). Mesenchymal stromal/stem cells and their extracellular vesicles application in acute and chronic inflammatory liver diseases: emphasizing on the anti-fibrotic and immunomodulatory mechanisms. *Frontiers in Immunology*.

[9] Tsuji K., Kitamura S., Wada J. (2020). Immunomodulatory and regenerative effects of mesenchymal stem cell-derived extracellular vesicles in renal diseases. *International Journal of Molecular Sciences*.

[10] Gao Z. -S., Zhang C. -J., Xia N. (2021). Berberine-loaded M2 macrophage-derived exosomes for spinal cord injury therapy. *Acta Biomaterialia*.

[11] Li F., Zhao L., Shi Y., Liang J. (2020). Edaravone-loaded macrophage-derived exosomes enhance neuroprotection in the rat permanent middle cerebral artery occlusion model of stroke. *Molecular pharmaceutics*.

[12] Herrmann I. K., Wood M. J. A., Fuhrmann G. (2021). Extracellular vesicles as a next-generation drug delivery platform. *Nature Nanotechnology*.

[13] Valadi H., Ekström K., Bossios A., Sjöstrand M. (2007). Exosome-mediated transfer of mRNAs and microRNAs is a novel mechanism of genetic exchange between cells. *Nature Cell Biology*.

[14] Shao M., Jin M., Xu S. (2020). Exosomes from long noncoding RNA-Gm37494-ADSCs repair sinal cord injury via shifting microglial M1/M2 polarization. *Inflammation*.

[15] Andaloussi S. E. L., Mäger I. O., Wood M. J. (2013). Extracellular vesicles: biology and emerging therapeutic opportunities. *Nature Reviews. Drug Discovery*.

[16] Yang Y., Hong Y., Cho E., Kim G. B., Kim I. S. (2018). Extracellular vesicles as a platform for membrane-associated therapeutic protein delivery. *Journal of Extracellular Vesicles*.

[17] He C., Zheng S., Luo Y., Wang B. (2018). Exosome theranostics: biology and translational medicine. *Theranostics*.

[18] Soltani S., Mansouri K., Emami Aleagha M. S. (2022). Extracellular vesicle therapy for type 1 diabetes. *Frontiers in Immunology*.

[19] Sedgwick A. E., D'Souza-Schorey C. (2018). The biology of extracellular microvesicles. *Traffic*.

[20] Quan C., Wang M., Chen H., Zhang H. (2021). Extracellular vesicles in acute respiratory distress syndrome: Recent developments from bench to bedside. *International Immunopharmacology*.

[21] Zhang L., Fan C., Hao W. (2021). NSCs migration promoted and drug delivered exosomes-collagen scaffold via a bio-specific peptide for one-step spinal cord injury repair. *Advanced Healthcare Materials*.

[22] Xu G., Ao R., Zhi Z., Jia J., Yu B. (2016). MiR-21 and miR-19b delivered by hMSC-derived EVs regulate the apoptosis and differentiation of neurons in patients with spinal cord injury. *Journal of cellular physiology*.

[23] Li R., Zhao K., Ruan Q., Meng C., Yin F. (2020). Bone marrow mesenchymal stem cell-derived exosomal microRNA-124-3p attenuates neurological damage in spinal cord ischemia-reperfusion injury by downregulating Ern1 and promoting M2 macrophage polarization. *Arthritis Research & Therapy*.

[24] Chen Y., Tian Z., He L. (2021). Exosomes derived from miR-26a-modified MSCs promote axonal regeneration via the PTEN/AKT/mTOR pathway following spinal cord injury. *Stem Cell Research & Therapy*.

[25] Shao M., Jin M., Xu S. (2020). Exosomes from long noncoding RNA-Gm37494-ADSCs repair Spinal cord injury via shifting microglial M1/M2 polarization. *Inflammation*.

[26] Luo Y., Xu T., Liu W. (2021). Exosomes derived from GIT1-overexpressing bone marrow mesenchymal stem cells promote traumatic spinal cord injury recovery in a rat model. *The International Journal of Neuroscience*.

[27] Jia Y., Lu T., Chen Q. (2021). Exosomes secreted from sonic hedgehog-modified bone mesenchymal stem cells facilitate the repair of rat spinal cord injuries. *Acta Neurochirurgica*.

[28] Kim H. Y., Kumar H., Jo M.-J. (2018). Therapeutic efficacy-potentiated and diseased rgoan-targeting nanovesicles derived from mesenchymal stem cells for spinal sord Injury treatment. *Nano Letters*.

[29] Lee J. -R., Kyung J. W., Kumar H., Kwon S. P. (2020). Targeted delivery of mesenchymal stem cell-derived nanovesicles for spinal cord injury treatment. *International Journal of Molecular Sciences*.

[30] Li L., Zhang Y., Mu J., Chen J. (2020). Transplantation of human mesenchymal stem-cell-derived exosomes immobilized in an adhesive hydrogel for effective treatment of spinal cord njury. *Nano letters*.

[31] Davidson S. M. (2021). Benefit of extracellular vesicles at the blood-brain barrier. *Arteriosclerosis, Thrombosis, and Vascular Biology*.

[32] Wang J., Chen S., Zhang W., Chen Y., Bihl J. C. (2020). Exosomes from miRNA-126-modified endothelial progenitor cells alleviate brain injury and promote functional recovery after stroke. *CNS Neuroscience & Therapeutics*.

[33] Zhao Y., Gan Y., Xu G., Hua K. (2020). Exosomes from MSCs overexpressing microRNA-223-3p attenuate cerebral ischemia through inhibiting microglial M1 polarization mediated inflammation. *Life Sciences*.

[34] Xin H., Liu Z., Buller B. (2021). MiR-17-92 enriched exosomes derived from multipotent mesenchymal stromal cells enhance axon-myelin remodeling and motor electrophysiological recovery after stroke,. *Official Journal of the International Society of Cerebral Blood Flow and Metabolism*.

[35] Chen W., Wang H., Zhu Z., Feng J. (2020). Exosome-shuttled circSHOC2 from IPASs regulates neuronal autophagy and ameliorates ischemic brain injury via the miR-7670-3p/SIRT1 axis. *Molecular therapy Nucleic Acids*.

[36] Yang J., Wu S., Hou L. (2020). Therapeutic effects of simultaneous delivery of nerve growth factor mRNA and protein via exosomes on cerebral ischemia. *Molecular therapy nucleic acids*.

[37] Yang H.-C., Zhang M., Wu R. (2020). C-C chemokine receptor type 2-overexpressing exosomes alleviated experimental post-stroke cognitive impairment by enhancing microglia/macrophage M2 polarization. *World Journal Stem Cells*.

[38] Li X., Zhang Y., Wang Y. (2020). Exosomes derived from CXCR4-overexpressing BMSC promoted activation of microvascular endothelial cells in cerebral ischemia/reperfusion injury. *Neural Plasticity*.

[39] Zhang H., Wu J., Wu J. (2019). Exosome-mediated targeted delivery of miR-210 for angiogenic therapy after cerebral ischemia in mice. *Journal of Nanobiotechnology*.

[40] Kim H. Y., Kim T. J., Kang L. (2020). Mesenchymal stem cell-derived magnetic extracellular nanovesicles for targeting and treatment of ischemic stroke. *Biomaterials*.

[41] Tian T., Zhang H. X., He C. P. (2018). Surface functionalized exosomes as targeted drug delivery vehicles for cerebral ischemia therapy. *Biomaterials*.

[42] Olanow C. W., Kieburtz K., Katz R. (2017). Clinical approaches to the development of a neuroprotective therapy for PD. *Experimental Neurology*.

[43] Izco M., Blesa J., Schleef M. (2019). Systemic exosomal delivery of shRNA minicircles prevents parkinsonian pathology. *Molecular Therapy*.

[44] Wu L., Zhou W., Lin L. (2021). Delivery of therapeutic oligonucleotides in nanoscale. *Bioactive Materials*.

[45] Tran P. H. L., Xiang D., Tran T. T. D. (2020). Exosomes and nanoengineering: a match made for precision therapeutics. *Advanced Materials*.

[46] Ren X., Zhao Y., Xue F. (2019). Exosomal DNA aptamer targeting *α*-synuclein aggregates reduced neuropathological deficits in a mouse parkinson's disease model. *Molecular Therapy Nucleic Acids*.

[47] Yang J., Luo S., Zhang J. (2021). Exosome-mediated delivery of antisense oligonucleotides targeting *α*-synuclein ameliorates the pathology in a mouse model of Parkinson's disease. *Neurobiology of Disease*.

[48] Haney M. J., Klyachko N. L., Zha Y. (2015). Exosomes as drug delivery vehicles for Parkinson's disease therapy. *Journal of controlled release*.

[49] Qu M., Lin Q., Huang L. (2018). Dopamine-loaded blood exosomes targeted to brain for better treatment of Parkinson's disease. *Journal of Controlled Release*.

[50] Liu L., Li Y., Peng H. (2020). Targeted exosome coating gene-chem nanocomplex as "nanoscavenger" for clearing *α*-synuclein and immune activation of Parkinson's disease. *Science advances*.

[51] Wang H., Sui H., Zheng Y. (2019). Curcumin-primed exosomes potently ameliorate cognitive function in AD mice by inhibiting hyperphosphorylation of the Tau protein through the AKT/GSK-3*β* pathway. *Nanoscale*.

[52] Wang H., Sui H., Zheng Y. (2020). Therapeutic effects of transplanted exosomes containing miR-29b to a rat model of Alzheimer's disease. *Frontiers in Neuroscience*.

[53] Blanc L., De Gassart A., Géminard C., Bette-Bobillo P., Vidal M. (2005). Exosome release by reticulocytes--an integral part of the red blood cell differentiation system. *Blood Cells, Molecules & Diseases*.

[54] Mulvihill J. J. E., Cunnane E. M., Ross A. M., Duskey J. T., Tosi G., Grabrucker A. M. (2020). Drug delivery across the blood-brain barrier: recent advances in the use of nanocarriers. *Nanomedicine*.

[55] Qi Y., Guo L., Jiang Y., Shi Y., Sui H., Zhao L. (2020). Brain delivery of quercetin-loaded exosomes improved cognitive function in AD mice by inhibiting phosphorylated tau-mediated neurofibrillary tangles. *Drug Delivery*.

[56] Cui G.-h., Guo H.-d., Li H. (2019). RVG-modified exosomes derived from mesenchymal stem cells rescue memory deficits by regulating inflammatory responses in a mouse model of Alzheimer's disease. *Immunity & Ageing*.

[57] Janas T., Sapoń K., Stowell M., Janas T. (2019). Selection of membrane RNA aptamers to amyloid beta peptide: implications for exosome-based antioxidant strategies. *International Journal of Molecular Sciences*.

[58] Gu S., Liu Y., Zou J. (2020). Retinal pigment epithelial cells secrete miR-202-5p-containing exosomes to protect against proliferative diabetic retinopathy. *Experimental Eye Research*.

[59] Cao X., Xue L. D., Di Y., Li T., Tian Y. J., Song Y. (2021). MSC-derived exosomal lncRNA SNHG7 suppresses endothelial-mesenchymal transition and tube formation in diabetic retinopathy via miR-34a-5p/XBP1 axis. *Life Sciences*.

[60] Li W., Jin L., Cui Y., Nie A., Xie N., Liang G. (2021). Bone marrow mesenchymal stem cells-induced exosomal microRNA-486-3p protects against diabetic retinopathy through TLR4/NF-*κ*B axis repression. *Journal of Endocrinological Investigation*.

[61] Li D., Zhang J., Liu Z., Gong Y., Zheng Z. (2021). Human umbilical cord mesenchymal stem cell-derived exosomal miR-27b attenuates subretinal fibrosis via suppressing epithelial-mesenchymal transition by targeting HOXC6. *Stem Cell Research & Therapy*.

[62] Dong X., Lei Y., Yu Z. (2021). Exosome-mediated delivery of an anti-angiogenic peptide inhibits pathological retinal angiogenesis. *Theranostics*.

[63] Tian Y., Zhang F., Qiu Y. (2021). Reduction of choroidal neovascularization via cleavable VEGF antibodies conjugated to exosomes derived from regulatory T cells. *Nature Biomedical Engineering*.

[64] Shi M.‐m., Yang Q.‐y., Monsel A. (2021). Preclinical efficacy and clinical safety of clinical-grade nebulized allogenic adipose mesenchymal stromal cells-derived extracellular vesicles. *Journal of Extracell Vesicles*.

[65] Jiang W., Luo F., Lu Q. (2016). The protective effect of Trillin LPS-induced acute lung injury by the regulations of inflammation and oxidative state. *Chemico-Biological Interactions*.

[66] Yi X., Wei X., Lv H. (2019). Exosomes derived from microRNA-30b-3p-overexpressing mesenchymal stem cells protect against lipopolysaccharide-induced acute lung injury by inhibiting SAA3. *Experimental Cell Research*.

[67] Song Y., Huang Z., Liu X. (2019). Platelet membrane-coated nanoparticle-mediated targeting delivery of Rapamycin blocks atherosclerotic plaque development and stabilizes plaque in apolipoprotein E-deficient (ApoE(-/-)) mice. *Nanomedicine: Nanotechnology, Biology, and Medicine*.

[68] Ma Q., Yao C., Shi H. (2021). Targeted delivery of dexamethasone in acute pneumonia. *Biomaterials Science*.

[69] Kim G. Y., Lee Y., Ha J., Han S., Lee M. (2021). Engineering exosomes for pulmonary delivery of peptides and drugs to inflammatory lung cells by inhalation. *Journal of Controlled Release*.

[70] Alzahrani F. A., Saadeldin I. M., Ahmad A. (2020). The potential use of mesenchymal stem cells and their derived exosomes as immunomodulatory agents for COVID-19 patients. *Stem Cells International*.

[71] Jamalkhah M., Asaadi Y., Azangou-Khyavy M. (2021). MSC-derived exosomes carrying a cocktail of exogenous interfering RNAs an unprecedented therapy in era of COVID-19 outbreak. *Journal of Translational Medicine*.

[72] Zhang M., Wang X., Han M. K., Collins J. F., Merlin D. (2017). Oral administration of ginger-derived nanolipids loaded with siRNA as a novel approach for efficient siRNA drug delivery to treat ulcerative colitis. *Nanomedicine*.

[73] Teng Y., Xu F., Zhang X. (2021). Plant-derived exosomal microRNAs inhibit lung inflammation induced by exosomes SARS-CoV-2 Nsp12. *Molecular Therapy*.

[74] Fu Y., Xiong S. (2021). Tagged extracellular vesicles with the RBD of the viral spike protein for delivery of antiviral agents against SARS-COV-2 infection. *Journal of Controlled Release : Official Journal of the Controlled Release Society*.

[75] Xie F., Su P., Pan T. (2021). Engineering extracellular vesicles enriched with palmitoylated ACE2 as COVID-19 therapy. *Advanced Materials*.

[76] Raghu G., Johnson W. C., Lockhart D., Mageto Y. (1999). Treatment of idiopathic pulmonary fibrosis with a new antifibrotic agent, pirfenidone: results of a prospective, open-label phase II study. *American Journal of Respiratory and Critical Care Medicine*.

[77] Myllärniemi M., Kaarteenaho R. (2015). Pharmacological treatment of idiopathic pulmonary fibrosis-preclinical and clinical studies of pirfenidone, nintedanib, and N-acetylcysteine. *European Clinical Respiratory Journal*.

[78] Kadota T., Fujita Y., Araya J. (2021). Human bronchial epithelial cell-derived extracellular vesicle therapy for pulmonary fibrosis via inhibition of TGF-*β*-WNT crosstalk. *Journal of Extracellular Vesicles*.

[79] Gao Y., Sun J., Dong C., Zhao M., Hu Y., Jin F. (2020). Extracellular vesicles derived from adipose mesenchymal stem cells alleviate PM2.5-induced lung injury and pulmonary fibrosis. *Medical Science Monitor: International Medical Journal of Experimental and Clinical Research*.

[80] Dinh P.-U. C., Paudel D., Brochu H. (2020). Inhalation of lung spheroid cell secretome and exosomes promotes lung repair in pulmonary fibrosis. *Nature Communications*.

[81] Guiot J., Cambier M., Boeckx A. (2020). Macrophage-derived exosomes attenuate fibrosis in airway epithelial cells through delivery of antifibrotic miR-142-3p. *Thorax*.

[82] Sun L., Fan M., Huang D. (2021). Clodronate-loaded liposomal and fibroblast-derived exosomal hybrid system for enhanced drug delivery to pulmonary fibrosis. *Biomaterials*.

[83] Mims J. W. (2015). Asthma: definitions and pathophysiology. *International Forum of Allergy & Rhinology*.

[84] Du Y.-m., Zhuansun Y.-x., Chen R., Lin L., Lin Y., Li J.-g. (2018). Mesenchymal stem cell exosomes promote immunosuppression of regulatory T cells in asthma. *Experimental Cell Research*.

[85] Ren J., Liu Y., Yao Y. (2021). Intranasal delivery of MSC-derived exosomes attenuates allergic asthma via expanding IL-10 producing lung interstitial macrophages in mice. *International Immunopharmacology*.

[86] Pei W., Li X., Bi R. (2021). Exosome membrane-modified M2 macrophages targeted nanomedicine: Treatment for allergic asthma. *Journal of Controlled Release*.

[87] Peng Y., Zhao J.-L., Peng Z.-Y., Xu W.-F., Yu G.-L. (2020). Exosomal miR-25-3p from mesenchymal stem cells alleviates myocardial infarction by targeting pro-apoptotic proteins and EZH2. *Cell Death & Disease*.

[88] Cai L., Chao G., Li W. (2020). Activated CD4+ T cells-derived exosomal miR-142-3p boosts post-ischemic ventricular remodeling by activating myofibroblast. *Aging*.

[89] Mao Q., Liang X. -L., Zhang C. -L., Pang Y. H., Lu Y. X. (2019). LncRNA KLF3-AS1 in human mesenchymal stem cell-derived exosomes ameliorates pyroptosis of cardiomyocytes and myocardial infarction through miR-138-5p/Sirt1 axis. *Stem Cell Research & Therapy*.

[90] Cheng H., Chang S., Xu R. (2020). Hypoxia-challenged MSC-derived exosomes deliver miR-210 to attenuate post-infarction cardiac apoptosis. *Stem Cell Research & Therapy*.

[91] Li Y., Zhou J., Zhang O. (2020). Bone marrow mesenchymal stem cells-derived exosomal microRNA-185 represses ventricular remolding of mice with myocardial infarction by inhibiting SOCS2. *International immunopharmacology*.

[92] Wei Z., Qiao S., Zhao J. (2019). MiRNA-181a over-expression in mesenchymal stem cell-derived exosomes influenced inflammatory response after myocardial ischemia-reperfusion injury. *Life sciences*.

[93] Ma J., Zhao Y., Sun L. (2017). Exosomes derived from Akt-modified human umbilical cord mesenchymal stem cells improve cardiac regeneration and promote angiogenesis via activating platelet-derived growth factor D. *Stem cells translational medicine*.

[94] Gong X. H., Liu H., Wang S. J., Liang S. W., Wang G. G. (2019). Exosomes derived from SDF1-overexpressing mesenchymal stem cells inhibit ischemic myocardial cell apoptosis and promote cardiac endothelial microvascular regeneration in mice with myocardial infarction. *Journal of Cellular Physiology*.

[95] Ni J., Liu X., Yin Y., Zhang P., Xu Y. W., Liu Z. (2019). Exosomes derived from TIMP2-modified human umbilical cord mesenchymal stem cells enhance the repair effect in rat model with myocardial infarction possibly by the Akt/Sfrp2 pathway. *Oxidative Medicine and Cellular Longevity*.

[96] Huang H., Xu Z., Qi Y. (2020). Exosomes from SIRT1-overexpressing ADSCs restore cardiac function by improving angiogenic function of EPCs. *Moleculat therapy nucleic acids*.

[97] Liu X., Li X., Zhu W. (2020). Exosomes from mesenchymal stem cells overexpressing MIF enhance myocardial repair. *Journal of Cellular Physiology*.

[98] Kang K., Ma R., Cai W. (2015). Exosomes secreted from CXCR4 overexpressing mesenchymal stem cells promote cardioprotection via Akt signaling pathway following myocardial infarction. *Stem Cells International*.

[99] Yu B., Kim H. W., Gong M. (2015). Exosomes secreted from GATA-4 overexpressing mesenchymal stem cells serve as a reservoir of anti-apoptotic microRNAs for cardioprotection. *International journal of cardiology*.

[100] Sun J., Shen H., Shao L. (2020). HIF-1*α* overexpression in mesenchymal stem cell-derived exosomes mediates cardioprotection in myocardial infarction by enhanced angiogenesis. *Stem Cell Research & Therapy*.

[101] Mentkowski K. I., Lang J. K. (2019). Exosomes engineered to express a cardiomyocyte binding peptide demonstrate improved cardiac retention in Vivo. *Scientific Reports*.

[102] Kim H., Yun N., Mun D. (2018). Cardiac-specific delivery by cardiac tissue-targeting peptide-expressing exosomes. *Biochemical and Biophysical Research Communications*.

[103] Wang X., Chen Y., Zhao Z. (2018). Engineered exosomes with ischemic myocardium-targeting peptide for targeted therapy in myocardial infarction. *Journal of the American Heart Association*.

[104] Yao C., Wu W., Tang H. (2020). Self-assembly of stem cell membrane-camouflaged nanocomplex for microRNA-mediated repair of myocardial infarction injury. *Biomaterials*.

[105] Lee J. R., Park B. W., Kim J. (2020). Nanovesicles derived from iron oxide nanoparticles-incorporated mesenchymal stem cells for cardiac repair. *Science Advances*.

[106] Liu S., Chen X., Bao L. (2020). Treatment of infarcted heart tissue via the capture and local delivery of circulating exosomes through antibody-conjugated magnetic nanoparticles. *Nature Biomedical Engineering*.

[107] Gallet R., Dawkins J., Valle J. (2017). Exosomes secreted by cardiosphere-derived cells reduce scarring, attenuate adverse remodelling, and improve function in acute and chronic porcine myocardial infarction. *European Heart Journal*.

[108] Liu B., Lee B. W., Nakanishi K. (2018). Cardiac recovery via extended cell-free delivery of extracellular vesicles secreted by cardiomyocytes derived from induced pluripotent stem cells. *Nature Biomedical Engineering*.

[109] Chen S., Zhou H., Zhang B., Hu Q. (2020). Exosomal miR-512-3p derived from mesenchymal stem cells inhibits oxidized low-density lipoprotein-induced vascular endothelial cells dysfunction via regulating Keap1. *Journal of Biochemical and Molecular Toxicology*.

[110] Yang W., Yin R., Zhu X. (2021). Mesenchymal stem-cell-derived exosomal miR-145 inhibits atherosclerosis by targeting JAM-A. *Molecular Therapy Nucleic Acids*.

[111] Wu G., Zhang J., Zhao Q. (2020). Molecularly engineered macrophage-derived exosomes with inflammation tropism and intrinsic heme biosynthesis for atherosclerosis treatment. *Angewandte Chemie*.

[112] Ko K. W., Yoo Y. I., Kim J. Y. (2020). Attenuation of tumor necrosis factor-*α* induced inflammation by umbilical cord-mesenchymal stem cell derived exosome-mimetic nanovesicles in endothelial cells. *Tissue engineering and regenerative medicine*.

[113] Lou G., Yang Y., Liu F. (2017). MiR-122 modification enhances the therapeutic efficacy of adipose tissue-derived mesenchymal stem cells against liver fibrosis. *Journal of Cellular and Molecular Medicine*.

[114] Qu Y., Zhang Q., Cai X. (2017). Exosomes derived from miR-181-5p-modified adipose-derived mesenchymal stem cells prevent liver fibrosis via autophagy activation. *Journal of Cellular and Molecular Medicine*.

[115] Tang M., Chen Y., Li B. (2021). Therapeutic targeting of STAT3 with small interference RNAs and antisense oligonucleotides embedded exosomes in liver fibrosis. *FASEB Journal*.

[116] Li Z., Zhou X., Wei M. (2019). In vitro and in vivo RNA inhibition by CD9-HuR functionalized exosomes encapsulated with miRNA or CRISPR/dCas9. *Nano Letters*.

[117] Li Z., Zhou X., Gao X. (2020). Fusion protein engineered exosomes for targeted degradation of specific RNAs in lysosomes: a proof-of-concept study. *Journal of Extracell Vesicles*.

[118] Sun C. K., Chen C. H., Chang C. L. (2017). Melatonin treatment enhances therapeutic effects of exosomes against acute liver ischemia-reperfusion injury. *American Journal of Translational Research*.

[119] Shao M., Xu Q., Wu Z. (2020). Exosomes derived from human umbilical cord mesenchymal stem cells ameliorate IL-6-induced acute liver injury through miR-455-3p. *Stem Cell Research & Therapy*.

[120] Wang G., Yuan J., Cai X. (2020). HucMSC-exosomes carrying miR-326 inhibit neddylation to relieve inflammatory bowel disease in mice. *Clinical and Translational Medicine*.

[121] Wang X., Zhang H., Yang H. (2018). Cell-derived exosomes as promising carriers for drug delivery and targeted therapy. *Current Cancer Drug Targets*.

[122] Sun D., Cao H., Yang L. (2020). MiR-200b in heme oxygenase-1-modified bone marrow mesenchymal stem cell-derived exosomes alleviates inflammatory injury of intestinal epithelial cells by targeting high mobility group box 3. *Cell Death & Disease*.

[123] Xiong J., Hu H., Guo R., Wang H., Jiang H. (2021). Mesenchymal stem cell exosomes as a new strategy for the treatment of diabetes complications. *Frontiers in Endocrinology*.

[124] Xu H., Du X., Xu J. (2020). Pancreatic *β* cell microRNA-26a alleviates type 2 diabetes by improving peripheral insulin sensitivity and preserving *β* cell function. *PLoS Biology*.

[125] Castaño C., Mirasierra M., Vallejo M., Novials A., Párrizas M. (2020). Delivery of muscle-derived exosomal miRNAs induced by HIIT improves insulin sensitivity through down-regulation of hepatic FoxO1 in mice. *Proceedings of the National Academy of Sciences of the United States of America*.

[126] Ying W., Gao H., Dos Reis F. C. G. (2021). MiR-690, an exosomal-derived miRNA from M2-polarized macrophages, improves insulin sensitivity in obese mice. *Cell Metabolism*.

[127] Zhuang M., Du D., Pu L. (2019). SPION-decorated exosome delivered BAY55-9837 targeting the pancreas through magnetism to improve the blood GLC response. *Small*.

[128] Zhang L., Zhao S., Zhu Y. (2020). Long noncoding RNA growth arrest-specific transcript 5 alleviates renal fibrosis in diabetic nephropathy by downregulating matrix metalloproteinase 9 through recruitment of enhancer of zeste homolog 2. *FASEB Journal*.

[129] Mao R., Shen J., Hu X. (2021). BMSCs-derived exosomal microRNA-let-7a plays a protective role in diabetic nephropathy via inhibition of USP22 expression. *Life Sciences*.

[130] Jin J., Wang Y., Zhao L., Zou W., Tan M., He Q. (2020). Exosomal miRNA-215-5p derived from adipose-derived stem cells attenuates epithelial-mesenchymal transition of podocytes by inhibiting ZEB2. *BioMed Research International*.

[131] Duan Y. R., Chen B. P., Chen F. (2021). Exosomal microRNA-16-5p from human urine-derived stem cells ameliorates diabetic nephropathy through protection of podocyte. *Journal of Cellular and Molecular Medicine*.

[132] Hao Y., Miao J., Liu W., Cai K., Huang X., Peng L. (2021). Mesenchymal stem cell-derived exosomes carry microRNA-125a to protect against diabetic nephropathy by targeting histone deacetylase 1 and downregulating endothelin-1. *Diabetes, Metabolic Syndrome and Obesity*.

[133] Fan B., Chopp M., Zhang Z. G., Liu X. S. (2021). Treatment of diabetic peripheral neuropathy with engineered mesenchymal stromal cell-derived exosomes enriched with microRNA-146a provide amplified therapeutic efficacy. *Experimental Neurology*.

[134] Singh A., Raghav A., Shiekh P. A., Kumar A. (2021). Transplantation of engineered exosomes derived from bone marrow mesenchymal stromal cells ameliorate diabetic peripheral neuropathy under electrical stimulation. *Bioactive Materials*.

[135] Ronco C., Bellomo R., Kellum J. A. (2019). Acute kidney injury. *Lancet*.

[136] Zhang Y., Wang J., Yang B. (2020). Transfer of microRNA-216a-5p from exosomes secreted by human urine-derived stem cells reduces renal ischemia/reperfusion injury. *Frontiers in Cell and Developmental Biology*.

[137] Cao J. Y., Wang B., Tang T. T. (2021). Exosomal miR-125b-5p deriving from mesenchymal stem cells promotes tubular repair by suppression of p53 in ischemic acute kidney injury. *Theranostics*.

[138] Viñas J. L., Burger D., Zimpelmann J. (2016). Transfer of microRNA-486-5p from human endothelial colony forming cell-derived exosomes reduces ischemic kidney injury. *Kidney International*.

[139] Yu W., Zeng H., Chen J. (2020). MiR-20a-5p is enriched in hypoxia-derived tubular exosomes and protects against acute tubular injury. *Clinical Science*.

[140] Grange C., Papadimitriou E., Dimuccio V. (2020). Urinary extracellular vesicles carrying klotho improve the recovery of renal function in an acute tubular injury model. *Molecular Therapy*.

[141] Wang B., Yao K., Huuskes B. M. (2016). Mesenchymal stem cells deliver exogenous microRNA-let7c via exosomes to attenuate renal fibrosis. *Molecular Therapy*.

[142] Wang H., Wang B., Zhang A. (2019). Exosome-mediated miR-29 transfer reduces muscle atrophy and kidney fibrosis in mice. *Molecular Therapy*.

[143] Tang T. T., Lv L. L., Wang B. (2019). Employing macrophage-derived microvesicle for kidney-targeted delivery of dexamethasone: an efficient therapeutic strategy against renal inflammation and fibrosis. *Theranostics*.

[144] Tang T. T., Wang B., Wu M. (2020). Extracellular vesicle-encapsulated IL-10 as novel nanotherapeutics against ischemic AKI. *Science Advances*.

[145] Yoon Y. M., Lee J. H., Song K. H., Noh H., Lee S. H. (2020). Melatonin-stimulated exosomes enhance the regenerative potential of chronic kidney disease-derived mesenchymal stem/stromal cells via cellular prion proteins. *Journal of Pineal Research*.

[146] Zhang C., Shang Y., Chen X. (2020). Supramolecular nanofibers containing arginine-glycine-aspartate (RGD) peptides boost therapeutic efficacy of extracellular vesicles in kidney repair. *ACS Nano*.

[147] Li Y., Jin D., Xie W. (2018). Mesenchymal stem cells-derived exosomes: a possible therapeutic strategy for osteoporosi. *Current Stem Cell Research & Therapy*.

[148] Zhi F., Ding Y., Wang R., Yang Y., Luo K., Hua F. (2021). Exosomal hsa_circ_0006859 is a potential biomarker for postmenopausal osteoporosis and enhances adipogenic versus osteogenic differentiation in human bone marrow mesenchymal stem cells by sponging miR-431-5p. *Stem Cell Research & Therapy*.

[149] Yang X., Yang J., Lei P., Wen T. (2019). LncRNA MALAT1 shuttled by bone marrow-derived mesenchymal stem cells-secreted exosomes alleviates osteoporosis through mediating microRNA-34c/SATB2 axis. *Aging*.

[150] Cao G., Meng X., Han X., Li J. (2020). Exosomes derived from circRNA Rtn4-modified BMSCs attenuate TNF-*α*-induced cytotoxicity and apoptosis in murine MC3T3-E1 cells by sponging miR-146a. *Bioscience Reports*.

[151] Zhang Y., Cao X., Li P. (2021). MicroRNA-935-modified bone marrow mesenchymal stem cells-derived exosomes enhance osteoblast proliferation and differentiation in osteoporotic rats. *Life Sciences*.

[152] Lu G. D., Cheng P., Liu T., Wang Z. (2020). BMSC-derived exosomal miR-29a promotes angiogenesis and osteogenesis. *Frontiers in cell and developmental biology*.

[153] Qiu M., Zhai S., Fu Q., Liu D. (2021). Bone marrow mesenchymal stem cells-derived exosomal microRNA-150-3p promotes osteoblast proliferation and differentiation in osteoporosis. *Human Gene Therapy*.

[154] Song H., Li X., Zhao Z. (2019). Reversal of osteoporotic activity by endothelial cell-secreted bone targeting and biocompatible exosomes. *Nano Letters*.

[155] Luo Z. W., Liu Y. W., Rao S. S. (2019). Aptamer-functionalized exosomes from bone marrow stromal cells target bone to promote bone regeneration. *Nanoscale*.

[156] Li C. -J., Cheng P., Liang M. -K. (2015). MicroRNA-188 regulates age-related switch between osteoblast and adipocyte differentiation. *The Journal of Clinical Investigation*.

[157] Itaka K., Ohba S., Miyata K. (2007). Bone regeneration by regulated in vivo gene transfer using biocompatible polyplex nanomicelles. *Molecular Therapy*.

[158] Hu Y., Li X., Zhang Q. (2021). Exosome-guided bone targeted delivery of antagomir-188 as an anabolic therapy for bone loss. *Bioactive materials*.

[159] Woolf A. D., Pfleger B. (2003). Burden of major musculoskeletal conditions. *Bull World Health Organ*.

[160] GBD 2017 Risk Factor Collaborators (2018). Global, regional, and national comparative risk assessment of 84 behavioural, environmental and occupational, and metabolic risks or clusters of risks for 195 countries and territories, 1990-2017: a systematic analysis for the Global Burden of Disease Study 2017. *Lancet*.

[161] Ni Z., Zhou S., Li S. (2020). Exosomes: roles and therapeutic potential in osteoarthritis. *Bone Research*.

[162] Skou S. T., Roos E. M., Laursen M. B. (2015). A randomized, controlled trial of total knee replacement. *The New England Journal of Medicine*.

[163] Zhang S., Chuah S. J., Lai R. C., Hui J. H. P., Lim S. K., Toh W. S. (2018). MSC exosomes mediate cartilage repair by enhancing proliferation, attenuating apoptosis and modulating immune reactivity. *Biomaterials*.

[164] Zhao Y., Xu J. (2018). Synovial fluid-derived exosomal lncRNA PCGEM1 as biomarker for the different stages of osteoarthritis. *International Orthopaedics*.

[165] Liu Y., Lin L., Zou R., Wen C., Wang Z., Lin F. (2018). MSC-derived exosomes promote proliferation and inhibit apoptosis of chondrocytes via lncRNA-KLF3-AS1/miR-206/GIT1 axis in osteoarthritis. *Cell Cycle*.

[166] Kolhe R., Hunter M., Liu S. (2017). Gender-specific differential expression of exosomal miRNA in synovial fluid of patients with osteoarthritis. *Scientific Report*.

[167] Mao G., Zhang Z., Hu S. (2018). Exosomes derived from miR-92a-3p-overexpressing human mesenchymal stem cells enhance chondrogenesis and suppress cartilage degradation via targeting WNT5A. *Stem Cell Research & Therapy*.

[168] He L., Chen Y., Ke Z. (2020). Exosomes derived from miRNA-210 overexpressing bone marrow mesenchymal stem cells protect lipopolysaccharide induced chondrocytes injury via the NF-kappaB pathway. *Gene*.

[169] Tao S. C., Yuan T., Zhang Y. L., Yin W. J., Guo S. C., Zhang C. Q. (2017). Exosomes derived from miR-140-5p-overexpressing human synovial mesenchymal stem cells enhance cartilage tissue regeneration and prevent osteoarthritis of the knee in a rat model. *Theranostics*.

[170] Wang Y., He S. H., Liang X., Zhang X. X., Li S. S., Li T. F. (2021). ATF4-modified serum exosomes derived from osteoarthritic mice inhibit osteoarthritis by inducing autophagy. *IUBMB Life*.

[171] Liang Y., Xu X., Li X. (2020). Chondrocyte-targeted microRNA delivery by engineered exosomes toward a cell-free osteoarthritis therapy. *ACS applied materials & interfaces*.

[172] Tuan R. S., Chen A. F., Klatt B. A. (2013). Cartilage regeneration. *The Journal of the American Academy of Orthopaedic Surgeons*.

[173] Xu X., Liang Y., Li X. (2021). Exosome-mediated delivery of kartogenin for chondrogenesis of synovial fluid-derived mesenchymal stem cells and cartilage regeneration. *Biomaterials*.

[174] Jiang S., Tian G., Yang Z. (2021). Enhancement of acellular cartilage matrix scaffold by Wharton’s jelly mesenchymal stem cell-derived exosomes to promote osteochondral regeneration. *Bioactive materials*.

[175] Rowland C. R., Glass K. A., Ettyreddy A. R. (2018). Regulation of decellularized tissue remodeling via scaffold-mediated lentiviral delivery in anatomically-shaped osteochondral constructs. *Biomaterials*.

[176] Hao Z. C., Lu J., Wang S. Z., Wu H., Zhang Y. T., Xu S. G. (2017). Stem cell-derived exosomes: a promising strategy for fracture healing. *Cell Proliferation*.

[177] Zhang L., Jiao G., Ren S. (2020). Exosomes from bone marrow mesenchymal stem cells enhance fracture healing through the promotion of osteogenesis and angiogenesis in a rat model of nonunion. *Stem Cell Research & Therapy*.

[178] Liu W., Li L., Rong Y. (2020). Hypoxic mesenchymal stem cell-derived exosomes promote bone fracture healing by the transfer of miR-126. *Acta Biomaterials*.

[179] Liu W., Li J., Cheng M. (2018). Zinc-modified sulfonated polyetheretherketone surface with immunomodulatory function for guiding cell fate and bone regeneration. *Advanced Science*.

[180] Xiong Y., Chen L., Yan C. (2020). M2 Macrophagy-derived exosomal miRNA-5106 induces bone mesenchymal stem cells towards osteoblastic fate by targeting salt-inducible kinase 2 and 3. *Journal of Nanobiotechnology*.

[181] Yan Y., Chen H., Zhang H. (2019). Vascularized 3D printed scaffolds for promoting bone regeneration. *Biomaterials*.

[182] Gao X., Ran N., Dong X. (2018). Anchor peptide captures, targets, and loads exosomes of diverse origins for diagnostics and therapy. *Science Translational Medicine*.

[183] Zha Y., Li Y., Lin T., Chen J., Zhang S., Wang J. (2021). Progenitor cell-derived exosomes endowed with VEGF plasmids enhance osteogenic induction and vascular remodeling in large segmental bone defects. *Theranostics*.

[184] Fan J., Lee C. S., Kim S., Chen C., Aghaloo T., Lee M. (2020). Generation of small RNA-modulated exosome mimetics for bone regeneration. *ACS Nano*.

[185] Zhai M., Zhu Y., Yang M., Mao C. (2020). human mesenchymal stem cell derived exosomes enhance cell-free bone regeneration by altering their mirnas profiles. *Advanced Science*.

[186] Wei F., Li M., Crawford R., Zhou Y., Xiao Y. (2019). Exosome-integrated titanium oxide nanotubes for targeted bone regeneration. *Acta Biomaterialia*.

[187] Chen Z., Bachhuka A., Han S. (2017). Tuning chemistry and topography of nanoengineered surfaces to manipulate immune response for bone regeneration applications. *ACS nano*.

[188] Sadowska J. M., Wei F., Guo J. (2019). The effect of biomimetic calcium deficient hydroxyapatite and sintered *β*-tricalcium phosphate on osteoimmune reaction and osteogenesis. *Acta Biomaterialia*.

[189] Chen L., Wang D., Peng F. (2019). Nanostructural surfaces with different elastic moduli regulate the immune response by stretching macrophages. *Nano letters*.

[190] Fan L., Guan P., Xiao C. (2021). Exosome-functionalized polyetheretherketone-based implant with immunomodulatory property for enhancing osseointegration. *Bioactive Materials*.

[191] Rogers R. G., Fournier M., Sanchez L. (2019). Disease-modifying bioactivity of intravenous cardiosphere-derived cells and exosomes in mdx mice. *JCI Insight*.

[192] Ran N., Gao X., Dong X. (2020). Effects of exosome-mediated delivery of myostatin propeptide on functional recovery of mdx mice. *Biomaterials*.

[193] Leng L., Dong X., Gao X. (2021). Exosome-mediated improvement in membrane integrity and muscle function in dystrophic mice. *Molecular Therapy*.

[194] Conceição M., Forcina L., Wiklander O. P. (2021). Engineered extracellular vesicle decoy receptor-mediated modulation of the IL6 trans-signalling pathway in muscle. *Biomaterials*.

[195] Liu S. J., Meng M. Y., Han S. (2021). Umbilical cord mesenchymal stem cell-derived exosomes ameliorate HaCaT cell photo-aging. *Rejuvenation Research*.

[196] Dalirfardouei R., Jamialahmadi K., Jafarian A. H., Mahdipour E. (2019). Promising effects of exosomes isolated from menstrual blood-derived mesenchymal stem cell on wound-healing process in diabetic mouse model. *Journal of Tissue Engineering and Regenerative Medicine*.

[197] Jiang L., Zhang Y., Liu T. (2020). Exosomes derived from TSG-6 modified mesenchymal stromal cells attenuate scar formation during wound healing. *Biochimie*.

[198] Li X., Xie X., Lian W. (2018). Exosomes from adipose-derived stem cells overexpressing Nrf2 accelerate cutaneous wound healing by promoting vascularization in a diabetic foot ulcer rat model. *Experimental & Molecular Medicine*.

[199] Lv Q., Deng J., Chen Y., Wang Y., Liu B., Liu J. (2020). Engineered human adipose stem-cell-derived exosomes loaded with miR-21-5p to promote diabetic cutaneous wound healing. *Molecular Pharmaceutics*.

[200] Liang G., Kan S., Zhu Y., Feng S., Feng W., Gao S. (2018). Engineered exosome-mediated delivery of functionally active miR-26a and its enhanced suppression effect in HepG2 cells. *International Journal of Nanomedicine*.

[201] Alvarez-Erviti L., Seow Y., Yin H., Betts C., Lakhal S., Wood M. J. (2011). Delivery of siRNA to the mouse brain by systemic injection of targeted exosomes. *Nature biotechnology*.

[202] Kooijmans S. A., Stremersch S., Braeckmans K. (2013). Electroporation-induced siRNA precipitation obscures the efficiency of siRNA loading into extracellular vesicles. *Journal of Controlled Release*.

[203] Wang C., Liang C., Wang R. (2019). The fabrication of a highly efficient self-healing hydrogel from natural biopolymers loaded with exosomes for the synergistic promotion of severe wound healing. *Biomaterials Science*.

[204] Zhou L., Xi Y., Chen M. (2018). A highly antibacterial polymeric hybrid micelle with efficiently targeted anticancer siRNA delivery and anti-infection in vitro/in vivo. *Nanoscale*.

[205] Annabi N., Rana D., Sani E. S. (2017). Engineering a sprayable and elastic hydrogel adhesive with antimicrobial properties for wound healing. *Biomaterials*.

[206] Wang M., Wang C., Chen M. (2019). Efficient angiogenesis-based diabetic wound healing/skin reconstruction through bioactive antibacterial adhesive ultraviolet shielding nanodressing with exosome release. *ACS Nano*.

[207] Xu N., Wang L., Guan J. (2018). Wound healing effects of a Curcuma zedoaria polysaccharide with platelet-rich plasma exosomes assembled on chitosan/silk hydrogel sponge in a diabetic rat model. *International journal of Biological Macromolecules*.

[208] Zhao D., Yu Z., Li Y., Wang Y., Li Q., Han D. (2020). GelMA combined with sustained release of HUVECs derived exosomes for promoting cutaneous wound healing and facilitating skin regeneration. *Journal of molecular histology*.

[209] Shiekh P. A., Singh A., Kumar A. (2020). Exosome laden oxygen releasing antioxidant and antibacterial cryogel wound dressing OxOBand alleviate diabetic and infectious wound healing. *Biomaterials*.

[210] Zhang K., Yu L., Li F. R. (2020). Topical Application of exosomes derived from human umbilical cord mesenchymal stem cells in combination with sponge spicules for treatment of photoaging. *International Journal of Nanomedicine*.

[211] Qian Z., Bai Y., Zhou J. (2020). A moisturizing chitosan-silk fibroin dressing with silver nanoparticles-adsorbed exosomes for repairing infected wounds. *Journal of materials chemistry*.

[212] Hohnholt M. C., Geppert M., Dringen R. (2011). Treatment with iron oxide nanoparticles induces ferritin synthesis but not oxidative stress in oligodendroglial cells. *Acta Biomaterialia*.

[213] Fan C. H., Cheng Y. H., Ting C. Y. (2016). Ultrasound/magnetic targeting with SPIO-DOX-microbubble complex for image-guided drug delivery in brain tumors. *Theranostics*.

[214] Li X., Wang Y., Shi L. (2020). Magnetic targeting enhances the cutaneous wound healing effects of human mesenchymal stem cell-derived iron oxide exosomes. *Journal of Nanobiotechnology*.

[215] Tao S. C., Guo S. C., Li M., Ke Q. F., Guo Y. P., Zhang C. Q. (2017). Chitosan wound dressings incorporating exosomes derived from microRNA-126-overexpressing synovium mesenchymal stem cells provide sustained release of exosomes and heal full-thickness skin defects in a diabetic rat model. *Stem cells Translational Medicine, Stem Cells Translational Medicine*.

[216] Wang C., Wang M., Xu T. (2021). Engineering bioactive self-healing antibacterial exosomes hydrogel for promoting chronic diabetic wound healing and complete skin regeneration. *Theranostics*.

[217] Su D., Tsai H. I., Xu Z. (2019). Exosomal PD-L1 functions as an immunosuppressant to promote wound healing. *Journal of Extracellular Vesicles*.

[218] Shafei S., Khanmohammadi M., Heidari R. (2020). Exosome loaded alginate hydrogel promotes tissue regeneration in full-thickness skin wounds: An in vivo study. *Journal of Biomedical Materials Research*.

[219] Sheller-Miller S., Radnaa E., Yoo J. K. (2021). Exosomal delivery of NF-*κ*B inhibitor delays LPS-induced preterm birth and modulates fetal immune cell profile in mouse models. *Science Advances*.

[220] Yim N., Ryu S. W., Choi K. (2016). Exosome engineering for efficient intracellular delivery of soluble proteins using optically reversible protein-protein interaction module. *Nature Communications*.

[221] Choi H., Kim Y., Mirzaaghasi A. (2020). Exosome-based delivery of super-repressor I*κ*B*α* relieves sepsis-associated organ damage and mortality. *Science Advances*.

[222] Lin J., Wang Z., Huang J. (2021). Microenvironment-protected exosome-hydrogel for facilitating endometrial regeneration, fertility restoration, and live birth of offspring. *Small*.

[223] Xiao Y., Tian J., Wu W. C. (2021). Targeting central nervous system extracellular vesicles enhanced triiodothyronine remyelination effect on experimental autoimmune encephalomyelitis. *Bioactive Materials*.

